# TRIM21 ameliorates hepatic glucose and lipid metabolic disorders in type 2 diabetes mellitus by ubiquitination of PEPCK1 and FASN

**DOI:** 10.1007/s00018-023-04820-w

**Published:** 2023-05-30

**Authors:** Kaini Zhang, Chen Yang, Xin Zhou, Jin Liang, Jianjin Guo, Min Li, Yi Zhang, Shulin Shao, Peng Sun, Kai Li, Jingjing Huang, Fang Chen, Xiubin Liang, Dongming Su

**Affiliations:** 1 Department of Pathophysiology, Nanjing Medical University, Nanjing, 211166 China; 2 Department of Pathology, Nanjing Medical University, Nanjing, 211166 China; 3 Key Laboratory of Human Functional Genomics of Jiangsu Province, Nanjing Medical University, Nanjing, 211166 China; 4 Department of General Medicine, Shanxi Bethune Hospital, Shanxi Academy of Medical Sciences, Tongji Shanxi Hospital, Third Hospital of Shanxi Medical University, Taiyuan, 030032 China; 5 Department of General Medicine, Tongji Hospital, Tongji Medical College, Huazhong University of Science and Technology, Wuhan, 430030 China; 6 Department of Pathology, Jiangsu Cancer Hospital, Jiangsu Institute of Cancer Research, Nanjing Medical University Affiliated Cancer Hospital, Nanjing, 211800 China; 7 Department of Laboratory, Nanjing Pukou Hospital of Traditional Chinese Medicine, Nanjing, 211800 China; 8 Department of Geriatrics, The Fourth Affiliated Hospital of Nanjing Medical University, Nanjing, 211166 China

**Keywords:** TRIM21, PEPCK1 stability, FASN stability, Ubiquitination, Hepatic steatosis, Insulin resistance

## Abstract

Hepatic glucose and lipid metabolism disorders promote the development and progression of type 2 diabetes mellitus (T2DM), yet the underlying mechanisms are not fully understood. Here, we identify tripartite motif-containing protein 21 (TRIM21), a class IV TRIM family member, as a pivotal regulator of hepatic metabolism in T2DM for the first time. Bioinformatic analysis suggests that TRIM21 expression is significantly reduced in T2DM patients. Intriguingly, in a mouse model of obese diabetes, TRIM21 expression is predominantly reduced in the liver rather than in other metabolic organs. It is further demonstrated that hepatic overexpression of TRIM21 significantly ameliorates glucose intolerance, insulin resistance, hepatic steatosis, and dyslipidemia in obese diabetic mice. In contrast, the knockdown of TRIM21 promotes glucose intolerance, insulin resistance, and triglyceride accumulation. Mechanistically, both phosphoenolpyruvate carboxykinase 1 (PEPCK1) and fatty acid synthase (FASN) are the hepatic targets of TRIM21. We revealed that TRIM21 promotes the degradation of PEPCK1 and FASN through a direct protein–protein interaction mediated K48-linked ubiquitination. Notably, overexpression of PEPCK1 and FASN essentially abolished the beneficial effects achieved by TRIM21 overexpression in obese diabetic mice. Overall, our data demonstrate that TRIM21 is a novel regulator of hepatic metabolic disorder, and suggest TRIM21 as a promising therapeutic target for T2DM.

**Supplementary Information:**

The online version contains supplementary material available at 10.1007/s00018-023-04820-w.

## Introduction

Type 2 diabetes mellitus is one of the fastest-growing global health concerns and has become a significant public health problem worldwide [1]. It is characterized by insulin resistance and abnormally increased hepatic glucose production (HGP). The liver is a crucial organ in regulating lipid and glucose metabolism. Ectopic lipid accumulation and excessive HGP from sustained gluconeogenesis lead to hepatic insulin resistance and glucose intolerance [2, 3]. It has been shown that the disorder of hepatic glucose and lipid metabolism promotes the development and progression of type 2 diabetes mellitus, whereas improving glucose and lipid metabolism can attenuate hyperglycemia and insulin resistance [4–7]. Although the underlying mechanisms remain incompletely transparent, previous studies from our group and others demonstrated a close association between the dysregulation of catalytic activities or expression levels of metabolic key enzymes in hepatocytes and the progression of type 2 diabetes mellitus [8–10]. In this regard, identifying novel factors that regulate the activities and expression of metabolic enzymes may facilitate the development of new therapeutic strategies for type 2 diabetes mellitus.

The E3 ubiquitin ligase of tripartite motif-containing protein 21 (TRIM21), also known as RNF81, Ro52, and SSA1, harbors the RING domain, a B-box domain (B2), the CC region, and the terminal PRY-SPRY domain. TRIM21 is involved in multiple biological processes, including cell proliferation, differentiation, apoptosis, and, in particular, innate and acquired immune response [11–13]. Animals with TRIM21 deficiency are highly susceptible to virus infection; in contrast, upregulation of TRIM21 protects mice against the virus attack [14]. The dysregulation of TRIM21 expression contributes to the pathogenesis of various human cancers. Previous studies of TRIM21 have mainly focused on its participation in cancer and autoimmune diseases [15, 16]. TRIM21 has either positive or negative regulatory roles in inflammation via the ubiquitination of family members of IRF (interferon regulatory factor) [17, 18]. In cancerous diseases, TRIM21 has been shown to facilitate tumor lipid metabolism via mediating ubiquitination and degradation of fatty acid synthase (FASN), one of the rate-limiting enzymes in the de novo lipogenesis pathway [19, 20]. Furthermore, genomic data from an NCBI GEO database shows that TRIM21 mRNA level is decreased in the blood of type 2 diabetes mellitus patients (GEO, accession number GSE26168). However, the role and mechanism of TRIM21 in hepatic glucose and lipid metabolism remain to be defined and elucidated.

In this study, TRIM21 was markedly downregulated in the livers of steatosis patients and obese diabetic mice. We mechanistically reveal that TRIM21 interacts with phosphoenolpyruvate carboxykinase 1 (PEPCK1), a rate-limiting gluconeogenic enzyme, and promotes its degradation by catalyzing K48-linked ubiquitination. Consistent with the findings in the tumor studies [21, 22], TRIM21 mediates FASN ubiquitination and degradation in hepatocytes. Moreover, we showed that TRIM21 overexpression ameliorated glucose intolerance, insulin resistance, hepatic steatosis, and dyslipidemia in obese diabetic mice, whereas its knockdown promoted glucose intolerance, insulin resistance, and triglyceride accumulation. Expectedly, overexpression of PEPCK1 and FASN essentially abolished the improvement of hepatic metabolic disorder by TRIM21 overexpression in obese diabetic mice. Our results suggest that TRIM21 is a crucial suppressor of hepatic glucose and lipid metabolism disorders and insulin resistance and thus may serve as a molecular target for treating type 2 diabetes mellitus.

## Materials and methods

### Human liver samples

Human liver samples, including non-steatosis and hepatitis, were collected from Jiangsu Cancer Hospital. Written informed consent was signed by the subjects or by the families of the liver donors. Procedures involving human samples were approved by Nanjing Medical University Review Board and were consistent with the principles outlined in the Declaration of Helsinki.

The non-steatotic control samples were collected from normal liver regions of nonalcoholic fatty liver disease (NAFLD)-free donors who received liver sections due to liver hemangioma or hepatic cyst. The steatotic liver samples were collected from nonalcoholic steatohepatitis (NASH) patients who underwent liver biopsy, resection, or transplantation. Two pathologists independently diagnosed hierarchical steatosis according to standard histological criteria established by the NASH Clinical Research Network [23].

### Animals and treatments

Genetically leptin deletion (*ob/ob*) mice (8 weeks old), diabetic leptin receptor-mutated (*db/db*) mice, and their respective control wild-type mice were purchased from the Model Animal Research Center of Nanjing University. They were fed a regular diet consisting of a standard lab chow diet. Dietary intervention with a high-fat diet (HFD) containing 60% kcal from fat (D12492; Research Diets) or chow diet (11.4% calories from fat) was conducted for 12 weeks from 1 month of age in C57BL/6J mice. All male mice were housed with 3 to 5 animals per cage, with a 12-h light/dark cycle and free access to water, at 23–26 °C. All animal studies were performed according to guidelines established by the Research Animal Care Committee of Nanjing Medical University, China (Permit Number: IACUC-NJMU 2008001).

To overexpress TRIM21 in C57BL/6J or *db/db* mice, adenovirus of TRIM21 (2 × 10^9^ pfu) was injected at the age of 8 weeks after standard chow (NC) diet administration according to our previously published protocol [8]. To knock down the expression of TRIM21 in C57BL/6J mice, adenoviral short hairpin (Adsh) TRIM21 (2 × 10^9^ pfu) was injected via tail vein injection. The mice underwent metabolic analysis on days 4 to 10 and were sacrificed on day 13 after an 18 h fast.

### Isolation, culture, and treatment of mouse primary hepatocytes

Primary hepatocytes were isolated from male C57BL/6J mice (8–10 weeks old) using liver perfusion according to our previously published protocol [8]. The viability of primary hepatocytes was routine ≥ 90%, as determined by trypan blue exclusion. The hepatocytes were plated in 12-well culture dishes pre-coated with collagen. We used adenoviral vectors to perform the gain and loss of function assays; the viruses were added at a dose of 1 × 10^7^ pfu/well in the cell culture medium.

### The glucose tolerance test (GTT), pyruvate tolerance test (PTT), and insulin tolerance test (ITT)

Blood glucose levels were measured using a Glucometer Elite monitor (Accu-Chek; Roche). GTTs were performed by intraperitoneal (i.p.) injection of d-glucose (2 g/kg) after overnight fasting. PTTs were performed by intraperitoneal (i.p.) injection of pyruvate (2 g/kg for wild-type mice, 1.5 g/kg for HFD or *db/db* mice) after overnight fasting. ITTs were performed by i.p. injection of 1 unit/kg insulin after 4 h of fasting. Blood glucose levels were recorded before and at 15, 30, 60, 90, and 120 min after injection. GTTs and ITTs were performed at 7 and 13 days after adenovirus injection.

### Serum and tissue biochemical analysis

Whole blood was collected by eye orbit. The concentrations of metabolic factors were measured with a triglyceride (TG) assay kit (Nanjing Jiancheng Bioengineering Institute, Cat#A110-1-1), a total cholesterol assay kit (Nanjing Jiancheng Bioengineering Institute, Cat#A110-1-1), an AST assay kit (Nanjing Jiancheng Bioengineering Institute, Cat#C010-2-1), an ALT assay kit (Nanjing Jiancheng Bioengineering Institute, Cat#C009-2-1) and an Ultra Sensitive Mouse Insulin ELISA kit (Crystal Chem, Cat#CCM-90082-1).

### Co-immunoprecipitation (Co-IP) assays and mass spectrometry analysis

Co-IP assays were performed as previously described [24]. The lysates were incubated with anti-TRIM21 antibody (Proteintech, Cat#12108-1-AP), anti-PEPCK1 antibody (Proteintech, Cat#16754-1-AP), anti-FASN antibody (Proteintech, Cat#10624-1-AP) or control IgG overnight with the protein A/G agarose beads. The complexes were washed three times with lysis buffer, and the immunoprecipitated proteins were eluted from the beads. The eluted proteins were detected by immunoblotting after separation by SDS-PAGE.

The mass spectrometry was performed on the precipitated protein of HepG2 cells transfected with Adenovirus-TRIM21 or empty vectors (Shanghai Genechem Co., Ltd). The peptides were subjected to a nanospray (NSI) source, followed by tandem mass spectrometry (MS/MS) on a Q ExactiveTM Plus (Thermo) instrument coupled online to an ultra-performance liquid chromatograph (UPLC). The resulting MS/MS data were processed using Proteome Discover 1.3. The Mass spectrometry analysis was performed at the PTM Bio-lab (China).

### Ubiquitination assays

Mouse primary hepatocytes were transfected with the indicated plasmids and lysed in 10% SDS lysis buffer, then denatured by heating at 95 °C for 10 min and IP lysis buffer (20 mM Tris–HCl, pH 7.4; 150 mM NaCl; 1 mM EDTA; and 1% NP-40) were added to the lysates. After sonication and centrifugation (12,000*g* for 10 min), the supernatants were incubated with anti-HA (Abmart, Cat#M2003S,) or anti-PEPCK1 antibody (Proteintech, Cat#16754-1-AP) as well as protein A/G agarose beads at 4 °C for 4 h. The beads were then washed with IP lysis buffer three times. Furthermore, the protein was boiled with SDS loading buffer after centrifugation for 10 min. Finally, western blotting was performed.

### Immunohistochemical and immunofluorescence staining

Human liver samples and mouse liver tissues were fixed overnight with 4% paraformaldehyde at 4 °C. The samples were dehydrated, embedded in paraffin, and sectioned into 3 µm thick transverse sections. The sections were dewaxed, treated with 3% H_2_O_2_ for 15 min, microwaved for 15 min to unmask antigens, washed in PBS containing Tween-20 (PBST), and then incubated with rabbit anti-TRIM21 (1:100 dilution) (Proteintech, Cat#16754-1-AP) overnight at 4 °C. After three washes with PBST, the sections were incubated with the secondary antibody for 30 min at 37 °C. The sections were then rinsed, and diaminobenzidine was added as a chromogen.

For immunofluorescence staining, primary hepatocytes were fixed in 4% paraformaldehyde for 25 min at room temperature and lysed with 0.1% Triton X-100 in PBS for 20 min. After being blocked with 1% BSA for 1.5 h at 37 °C, the cells were incubated with mouse anti-TRIM21 antibody (Proteintech, Cat#67736-1-Ig), and rabbit anti-PEPCK1 antibody or rabbit anti-FASN antibody at 4 °C overnight. After three washes with PBS, the cells were incubated with the corresponding secondary antibodies for 1 h at 37 °C. The cells were stained with 4′,6-diamidino-2-phenylindole (DAPI) for 2 min and washed with PBS. All images were obtained using an Olympus confocal microscope and processed using Olympus FV1000 software.

### Western blotting analysis

To perform western blotting analysis, tissues and cells were isolated and lysed with RIPA lysis buffer. Total protein was quantified using a BCA kit (Thermo Fisher Scientific; Waltham, MA, USA; Cat#23225). Then, equal quantities of the indicated protein were separated by 10% SDS-PAGE and transferred to PVDF membranes (IPVH00010; Millipore; Billerica, MA, USA). After being blocked with 5% skim milk in TBST, the PVDF membranes with proteins were incubated with the indicated primary antibodies against TRIM21 (1:1000 dilution, Abcam, Cat#ab207728), PEPCK1 (1:2000 dilution, Proteintech, Cat#16754-1-Ap), FASN (1:1000 dilution, Cat# 10624-2-Ap) overnight at 4 °C and then incubated with HRP-conjugated secondary antibodies. The proteins were then detected using an ECL kit (170-5061; Bio-Rad; Hercules, CA, USA) and visualized in a ChemiDoc MP Imaging System (Bio-Rad; Hercules, CA, USA). The expression of the target proteins was normalized to the expression of the loading control, β-Actin or Tubulin.

### Quantitative RT-PCR analysis

Total RNA was extracted from liver tissues and cells by RNA-easy Isolation Reagent (Vazyme, Cat#R701). Complimentary DNA (cDNA) was obtained by reverse transcription of 0.5 mg RNA using a reverse transcription and cDNA synthesis kit. Quantitative real-time PCR analysis was performed with Cham Q SYBR qPCR Master Mix (High ROX premised) (Vazyme, Cat#Q341) and analyzed using a Roche Real-Time PCR System. Expression levels were determined using the relative standard curve method and normalized to the housekeeping gene β-Actin.

### Determination of total intracellular triglyceride amounts

Mouse primary hepatocytes were plated in six-well plates in Dulbecco’s modified Eagle’s medium (DMEM) medium containing 10% FBS. Intracellular triglyceride (TG) contents were measured using an Adipogenesis Assay Kit (Sigma-Aldrich, Cat#MAK040) according to the manufacturer’s instructions and normalized to total protein concentrations. Intracellular TG levels were expressed in nmol/μg protein.

### Quantification and statistical analysis

All of the experiments above were performed at least three times independently. The results are presented as means ± SEM. Significant differences were assessed using a two-tailed Student’s *t* test or one-way ANOVA followed by the Student–Newman–Keuls (SNK) test. A value of *P* < 0.05 was considered statistically significant.

## Results

### Hepatic TRIM21 expression was reduced in steatosis patients and obese diabetic mice

**Fig. 1:**
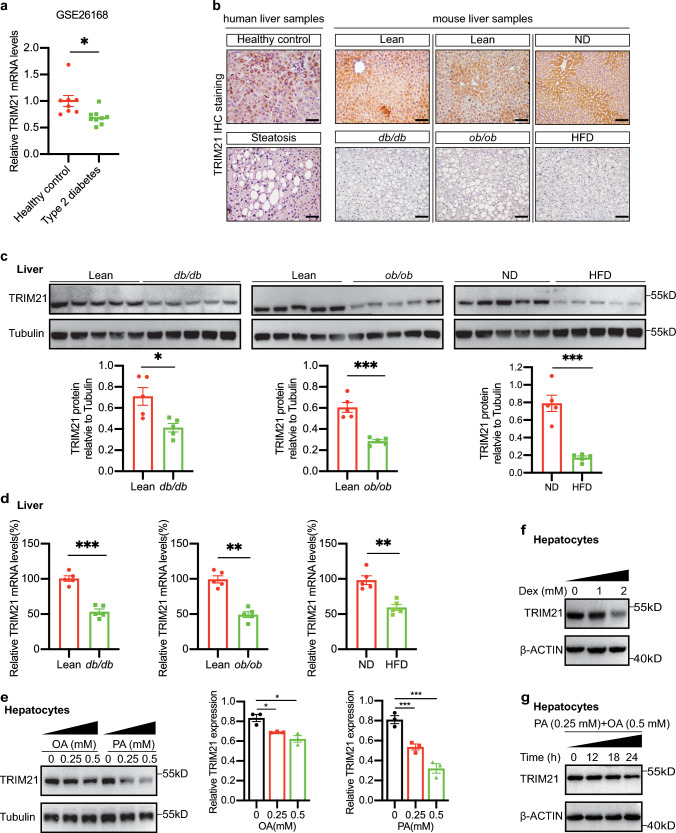
Hepatic TRIM21 expression was reduced in steatosis patients and obese diabetic mice. **a** The relative TRIM21 mRNA levels in the blood of healthy control and type 2 diabetes subjects from a GEO dataset (GSE26168). **b** Immunohistochemical staining of livers obtained from patients with non-steatosis and steatosis was performed for TRIM21 (left panel). Immunohistochemistry staining showed the expression level of TRIM21 in liver sections from *db/db* mice, *ob/ob* mice, and HFD-fed mice (right panel). Scale bars, 100 μm. *n* = 3 mice for each genotype. **c**, **d** The protein and mRNA levels of TRIM21 were measured in the livers of male *db/db* mice, *ob/ob* mice, or HFD-fed mice by western blotting analysis and quantitative real-time PCR assay. The comparisons are made with respective age-matched and sex-matched controls. Quantification data was shown in the lower panel. Data are presented as mean ± SEM. *P < 0.05, **P < 0.01, ***P < 0.001 vs. Lean. The data are normalized to tubulin or β-actin (*n* = 5 for all groups). **e**, **f** The expression of TRIM21 was detected in mouse primary hepatocytes treated with different concentrations of palmitic acid, oleic acid, or dexamethasone. Quantification data was shown in the right panel. Data are presented as mean ± SEM. *P < 0.05, **P < 0.01, ***P < 0.001 vs. control. The data are normalized to tubulin (*n* = 3 for all groups). **g** The expression of TRIM21 was detected in mouse primary hepatocytes treated with palmitic acid and oleic acid at different times. *PA* palmitic acid, *OA* oleic acid, *Dex* dexamethasone

To investigate whether TRIM21 expression is associated with human metabolic disorders, we first utilized bioinformatic analysis to observe the TRIM21 expression in type 2 diabetic patients. The data from the GEO dataset (https://www.ncbi.nlm.nih.gov/geo/, GSE26168) showed that TRIM21 mRNA levels were lower in the blood of type 2 diabetes mellitus patients (Fig. 1a). It is known that TRIM21 is expressed in numerous organs, however, the relative importance of organ-specific TRIM21 in type 2 diabetes mellitus has not been investigated. We found that the liver exhibited the highest TRIM21 expression compared with other metabolic organs, including epididymal white adipose tissue, brown adipose tissue, pancreatic islets, and skeletal muscle (data not shown). Furthermore, we measured TRIM21 expression in the livers of patients with steatosis. As shown by immunohistochemistry (IHC) staining, TRIM21 expression in steatosis patients' livers was significantly lower than in non-steatosis livers (Fig. 1b, left panel). Similar results were obtained in the liver sections of *db/db* mice, *ob/ob* mice and HFD-fed mice (Fig. 1b, right panel). This conclusion was also confirmed by western blot (Fig. 1c, upper panel, quantified in Fig. 1c, lower panel) and quantitative real-time PCR (Fig. 1d) analyses of liver tissues obtained from obese diabetic mice (*ob/ob* mice, *db/db* mice, and HFD-fed mice).

To further investigate the potential link between TRIM21 and liver glycolipid metabolism by mimicking hepatic steatosis in vitro, mouse primary hepatocytes were exposed to oleic acid (a monounsaturated fatty acid), palmitic acid (a saturated fatty acid), and dexamethasone. Cell lysates from primary hepatocytes were tested by western blotting for protein expressions of TRIM21. Consistently, TRIM21 expression was significantly reduced in mouse primary hepatocytes after oleic acid (OA), palmitic acid (PA), or dexamethasone (Dex) challenges (Fig. 1e, f). In addition, TRIM21 expression was dramatically suppressed by both PA (0.25 mM) and OA (0.5 mM) treatments in a time-dependent manner (Fig. 1g). These data suggested a potential regulatory role of TRIM21 on hepatic glucose and lipid metabolism.

### Ectopic expression of hepatic TRIM21 attenuates glucose intolerance and insulin resistance in obese diabetic mice

**Fig. 2:**
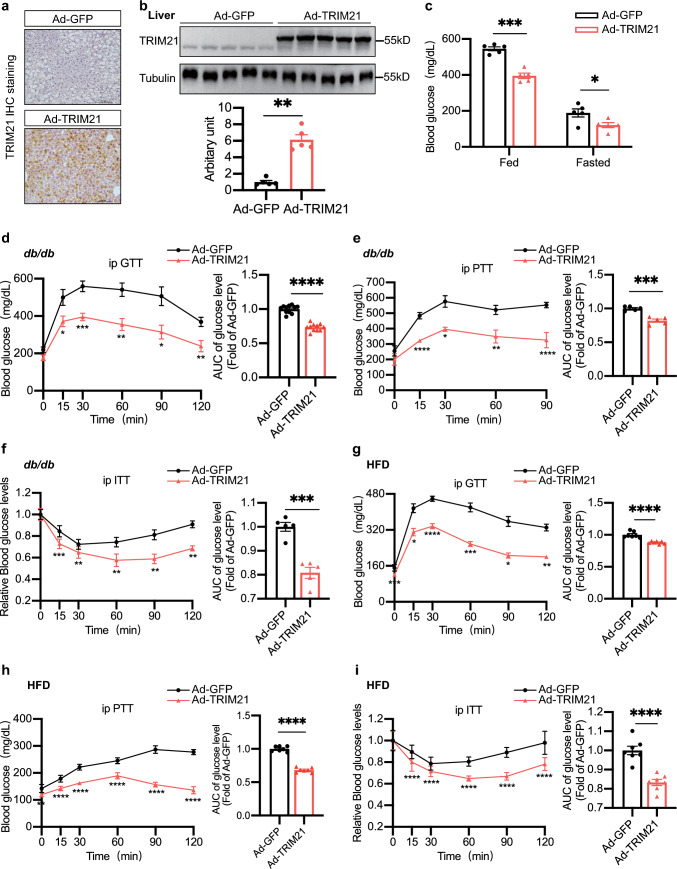
Ectopic expression of hepatic TRIM21 attenuates glucose intolerance and insulin resistance in obese diabetic mice. The male *db/db* mice (8–12 weeks old) were injected with adenovirus-GFP (Ad-GFP) or Ad-TRIM21 through the tail vein. Measurements were performed at 7 days post-injection. **a** Immunohistochemistry staining showed the expression level of TRIM21 in liver sections from adenovirus-injected *db/db* mice. Scale bars, 100 μm. *n* = 5 mice for each genotype. **b** Western blot assay showed the efficacy of adenovirus-mediated TRIM21 overexpression (upper panel, Western blot; lower panel, quantitative measurement of TRIM21 protein relative to tubulin). **c** Blood glucose levels were monitored at day 7 (fed) and day 8 (fast) after the adenovirus injection. **d** Glucose tolerance test (GTT) at day 7. **e** Pyruvate tolerance test (PTT) at day 10. **f** Insulin tolerance test (ITT) at day 13. The area under the curve (AUC) of GTT, PTT, and ITT was shown (right panel) (*n* = 5–11 mice for each genotype). **g**–**i** C57BL/6J mice fed HFD for 12 weeks were injected with Ad-GFP or Ad-TRIM21 via tail vein injection. Seven days after injection, GTT (**g**), PTT (**h**), and ITT (**i**) were measured as indicated. The area under the curve (AUC) was shown (right panel) (*n* = 7 mice for each genotype). Data were presented as mean ± SEM. *P < 0.05, **P < 0.01, ***P < 0.001, ****P < 0.0001 vs. Ad-GFP group

To confirm the potentially protective role of TRIM21 in the pathogenesis of steatosis and related metabolic disorders, we used an adenoviral approach to achieve TRIM21 overexpression in mouse liver in vivo. We achieved more than 85% transduction efficiency in the hepatocytes of *db/db* mice and HFD mice 7 days later by administering these adenoviral particles via tail vein injection (Fig. 2a, b, quantified in Fig. 2b, lower panel). Meanwhile, TRIM21 adenoviral particle injection did not alter TRIM21 expression in other major metabolic organs such as adipose tissues, skeletal muscle, and pancreas, among others (data not shown). Body weight (Supplementary Fig. 1a) and food intake (Supplementary Fig. 1b) were not affected after the adenovirus-TRIM21 (Ad-TRIM21) injection. Interestingly, Ad-TRIM21-injected *db/db* mice displayed a significant decrease in blood glucose levels (Fig. 2c). In addition, glucose intolerance, pyruvate tolerance, and insulin resistance were markedly improved by hepatic overexpression of TRIM21, evidenced by glucose tolerance test (GTT), pyruvate tolerance test (PTT), insulin tolerance test (ITT) results, indicating that TRIM21 increased insulin sensitivity (Fig. 2d–f, left panels). The area under the curve (AUC) of the GTT, PTT, and ITT in TRIM21 overexpression mice was also decreased (Fig. 2d–f, right panels). However, there was no difference in insulin secretion after the adenovirus-TRIM21 (Ad-TRIM21) injection (Supplementary Fig. 1c). Similar results were observed in HFD mice (Fig. 2g–i). Together, these results demonstrated that overexpression of hepatic TRIM21 attenuates glucose intolerance and insulin resistance in obese diabetic mice.

### Ectopic expression of TRIM21 alleviated hepatic steatosis and dyslipidemia in obese diabetic mice and hepatocytes

**Fig. 3:**
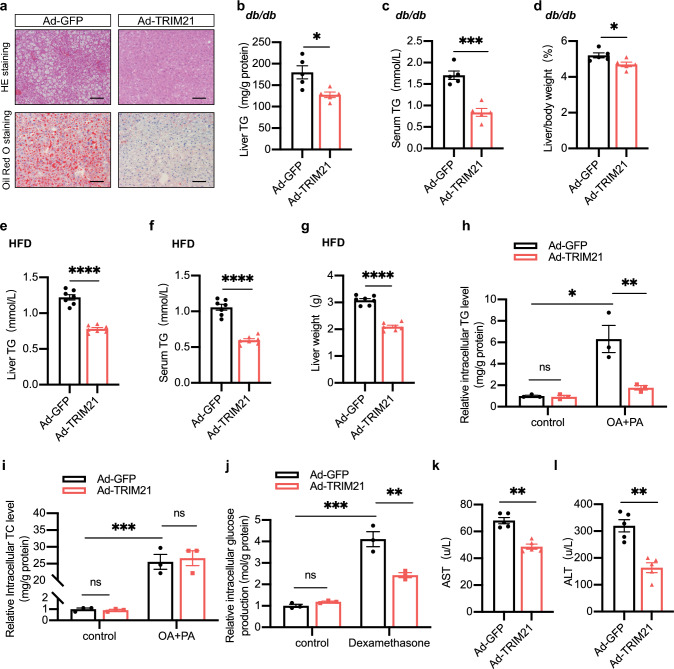
TRIM21 alleviated hepatic steatosis and dyslipidemia in obese diabetic mice. Male *db/db* mice (eight weeks old) were injected with Ad-GFP or Ad-TRIM21 via tail vein injection. **a** Representative micrographs showed hepatic steatosis in HE and Oil Red O staining sections from the adenovirus-injected *db/db* mice at day 7 after injection. Scale bar, 100 μm. **b**–**d** Liver TG levels (**b**), serum TG levels (**c**), and relative liver weight (**d**) were measured as indicated. *P < 0.05, **P < 0.01, ***P < 0.001 vs. Ad-GFP group. *n* = 5 mice for each group. **e**–**g** C57BL/6J mice fed HFD for 12 weeks were injected with Ad-GFP or Ad-TRIM21 via tail vein injection. Liver TG levels (**e**), serum TG levels (**f**), and liver weight (**g**) were measured as indicated. ****P < 0.0001 vs. Ad-GFP group. *n* = 7 mice for each group. **h**, **i** Mouse primary hepatocytes were infected with Ad-GFP or Ad-TRIM21 for 24 h, then treated with palmitic acid (PA) and oleic acid (OA) for another 24 h. PA, 0.25 mM. OA, 0.5 mM. Intracellular TG levels (**h**) and intracellular TC levels (**i**) were measured at the indicated treatment. *P < 0.05, **P < 0.01, ***P < 0.001. *n* = 3 for each group. **j** Mouse primary hepatocytes were infected with Ad-GFP or Ad-TRIM21 for 24 h, then treated with dexamethasone (2 mM) for another 24 h. *P < 0.05, **P < 0.01, ***P < 0.001. *n* = 3 for each group. **k** Serum ALT level. **P < 0.01. *n* = 5 mice for each group. **l** Serum AST level. **P < 0.01. *n* = 5 mice for each group. Data are presented as mean ± SEM

Next, we assessed the effect of TRIM21 in the development of hepatic steatosis and dyslipidemia. Liver tissues stained with hematoxylin–eosin (HE) staining and Oil Red O staining analyses indicated a significant decrease in hepatic lipid deposition after TRIM21 overexpression in *db/db* mice compared with control mice (Fig. 3a), which correlated with the decrease of hepatic triglyceride (TG) content (Fig. 3b). Serum TG levels were also strikingly decreased (≈ 50%) by hepatic overexpression of TRIM21 (Fig. 3c). Remarkably, the relative liver weight was significantly decreased in Ad-TRIM21-injected *db/db* mice relative to control mice (Fig. 3d). Similar results were also observed in HFD-diet mice (Fig. 3e–g). According to quantitative real-time PCR analysis, hepatic TRIM21 repressed the expression levels of several lipogenic genes, while several β-oxidation and fatty acid uptake genes were unchanged (Supplementary Fig. 2). In mouse primary hepatocytes, ectopic TRIM21 expression attenuated intracellular TG accumulation in hepatocytes exposed to the combination of PA (0.25 mM) and OA (0.5 mM), was consistent with the in-vivo data (Fig. 3h). However, total cholesterol (TC) was not significantly changed (Fig. 3i). Consistently, intracellular glucose production was inhibited in TRIM21 overexpression mouse primary hepatocytes exposed to dexamethasone (Fig. 3j).

Besides various degrees of steatosis, hepatocellular damage is another essential feature of hepatic glucose and lipid metabolism disorders. Interestingly, we noted that adenoviral TRIM21 overexpression attenuated lipogenic liver injury, as evidenced by the decreased ALT (Fig. 3k) and AST (Fig. 3l) levels in *db/db* mice in comparison with control adenovirus infection in those mice.

Overall, overexpression of hepatic TRIM21 attenuated lipid accumulation and dyslipidemia in obese diabetic mice.

### Knockdown of hepatic TRIM21 promoted liver steatosis and insulin resistance

**Fig. 4:**
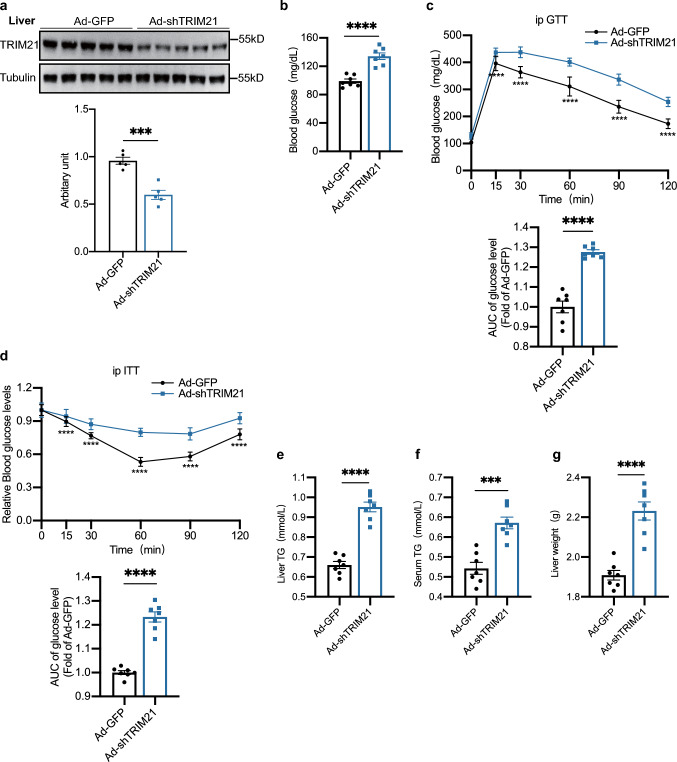
Knockdown of hepatic TRIM21 promoted liver steatosis and insulin resistance. C57BL/6J mice fed a HFD for 12 weeks were injected with Ad-GFP or Ad-shTRIM21 via tail vein injection. Measurements were performed at 7 days post-injection. **a** Western blot assay shows the efficacy of adenoviral short hairpin-mediated TRIM21 knockdown (upper, Western blot; lower, quantitative measurement of TRIM21 protein relative to Tubulin). Fasting glucose level (**b**), GTT (**c**), ITT (**d**), liver TG levels (**e**), serum TG levels (**f**), and liver weight (**g**) were measured as indicated (*n* = 7 mice per group). Data are presented as mean ± SEM. *P < 0.05, **P < 0.01, ***P < 0.001, ****P < 0.0001 vs. Ad-GFP group

Then, we injected Ad-shTRIM21 through the tail vein to knock down TRIM21 in the C57BL/6J mouse livers. Efficiency tests showed a significant reduction of TRIM21 expression in the liver samples 7 days after the tail vein injection (Fig. 4a, quantified in Fig. 4a, lower panel). By contrast, on days 4 and 7 after the infection, Ad-shTRIM21-injected mice displayed fasting hyperglycemia, glucose intolerance, and insulin resistance compared with Ad-GFP-injected mice (Fig. 4b–d). In GTT and ITT, AUC was higher in the Ad-shTRIM21 groups relative to the Ad-GFP groups (Fig. 4c, d, lower panels). As expected, liver TG and serum TG levels were significantly higher in Ad-shTRIM21-injected mice than in Ad-GFP-injected mice (Fig. 4e, f). Similarly, liver weight was higher in Ad-shTRIM21-injected mice (Fig. 4g). However, body weight (Supplementary Fig. 1d) and food intake (Supplementary Fig. 1e) were not affected by Ad-shTRIM21 injection. These findings verified that loss of hepatic TRIM21 promoted liver steatosis and insulin resistance.

### TRIM21 directly interacted with PEPCK1 and FASN

**Fig. 5:**
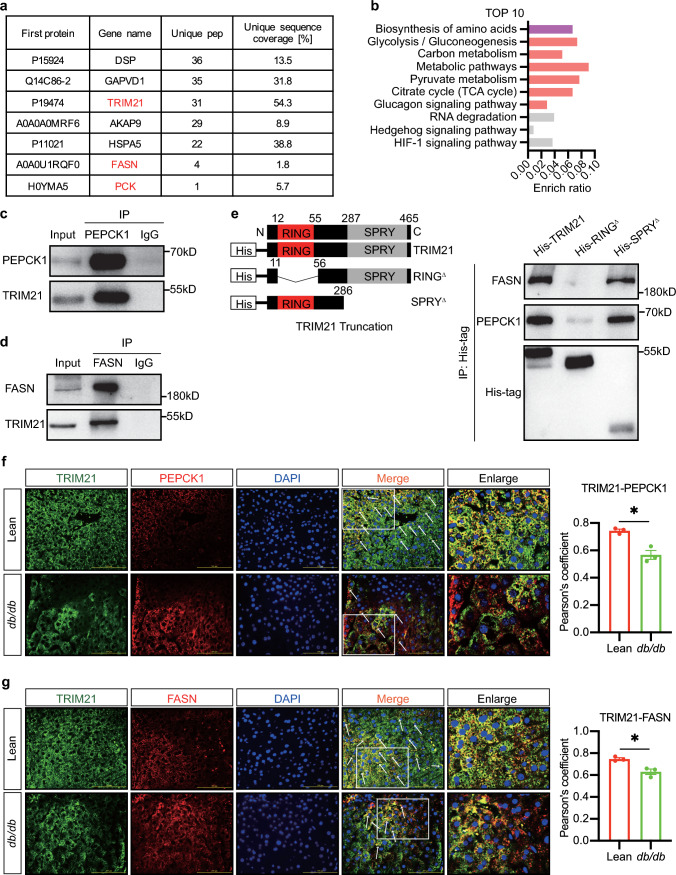
TRIM21 directly interacted with PEPCK1 and FASN. **a** Seven candidates were shown in TRIM21 co-IP-based LC–MS/MS analysis using HepG2 immortalized human hepatoma cell-line. **b** Visualization of gene-list enrichment results. The barplot was the top 10 terms. The length of the bar represents the enrichment ratio. The color of the bar represents different clusters. **c**, **d** Co-IP assay using TRIM21 antibody, PEPCK1 antibody, or FASN antibody in mouse primary hepatocytes without exogenous TRIM21 expression. **e** Mouse primary hepatocytes were transfected with TRIM21 WT or the indicated truncation mutants for 48 h and incubated with the proteasome inhibitor MG132 (10 μmol/L) for 4 h before being lysed. Co-immunoprecipitation (IP) was performed to detect the interaction between TRIM21 and PEPCK1/FASN. **f**, **g** Immunofluorescence staining of TRIM21 (green) and PEPCK1/ FASN (red) in control or *db/db* mice liver sections. White arrows indicate the co-localization of TRIM21 and PEPCK1/ FASN in hepatocytes. Scale bar, 100 μm. Pearson’s coefficient was shown (right panels). *P < 0.05 vs. Lean. *n* = 3

To study the molecular mechanism of TRIM21 on hepatic glucose and lipid metabolism, we next sought to identify the proteins interacting with TRIM21 using a co-immunoprecipitation (co-IP) based liquid chromatography-tandem mass spectrometry (LC–MS/MS) method in Ad-TRIM21- or Ad-GFP-infected HepG2 immortalized human hepatoma cell-line. We found that more than 299 proteins might be candidate substrates of TRIM21 (Supplementary Table 1). Among these, phosphoenolpyruvate carboxykinase-1 (PEPCK1) and fatty acid synthase (FASN) were identified to be abundantly pulled down by TRIM21, especially with ectopic TRIM21 expression (Fig. 5a). Then, a total of genes was identified and submitted to the gene-list enrichment using the KEGG pathway database (http://bioinfo.org/kobas). The top 10 terms with calculated enriched *P*-values (*P* < 0.05) were then visualized using cirFunMap (correlation > 0.35). Interestingly, the top 10 terms focused on metabolism-related pathways (Fig. 5b). Meanwhile, our findings exhibit a significant upregulation of PEPCK1 and FASN expression in the liver of HFD mice compared to their controls, underscoring the pivotal role of PEPCK1 and FASN on hepatic metabolic disorders (Supplementary Fig. 5a). We then conducted co-IP experiments to examine the interaction between TRIM21 and PEPCK1, as well as the interaction between TRIM21 and FASN. In mouse primary hepatocytes, we used antibodies against PEPCK1 or FASN to isolate proteins from mouse primary hepatocytes. The isolated proteins were then examined by western blotting to detect binding signals of TRIM21. IgG was used as a control which yielded no positive signal in immunoprecipitations (IPs). The binding signals of PEPCK1 or FASN with TRIM21 were detected by anti-TRIM21 antibody, and subsequently blotting with PEPCK1 or FASN antibody. Consistently, co-IP assays confirmed physical TRIM21 bindings with PEPCK1 and FASN in mouse primary hepatocytes (Fig. 5c, d).

As mentioned, the E3 ubiquitin ligase of TRIM21 harbors the RING domain, a B-box domain (B2), the CC region, and the terminal PRY-SPRY domain [11]. The E3 ubiquitin ligase has been previously shown to bind substrate through its RING domain, which is responsible for ubiquitination and degradation. To validate this, we expressed and purified the recombinant His-TRIM21 deletion mutant (lacking residues 12–55 located in the RING domain), which we tentatively named His-RING^Δ^, and tested its binding activity in vitro. In addition, the recombinant His-TRIM21 deletion mutant (lacking residues 286–465 located in the SPRY domain) was constructed, which we tentatively named His-SPRY^Δ^ (Fig. 5e, left panel). We verified this speculation by transfecting TRIM21 WT (wild type) plasmids, His-RING^Δ^ plasmids, and His-SPRY^Δ^ plasmids into mouse primary hepatocytes. His-tag was immunoprecipitated and the protein expression of FASN, PEPCK1 and His-tag was observed by western blotting. As shown in Fig. 5e, right panel, PEPCK1 co-precipitated with TRIM21 WT and His-SPRY^Δ^, indicating that the RING-finger domain contributes to binding to PEPCK1. Similar results were also observed between FASN and TRIM21.

Immunofluorescence staining revealed the co-localization of TRIM21/PEPCK1 and TRIM21/FASN in the cytoplasm of control mouse hepatocytes (Fig. 5f, g, upper panels), in agreement with the co-IP analysis; however, in the *db/db* mouse hepatocytes, this co-localization was decreased (Fig. 5f, g, lower panels). Pearson’s coefficient is shown in Fig. 5f, g, right panels. These data demonstrated that TRIM21 directly interacted with PEPCK1 and FASN.

### TRIM21 ubiquitinated and degraded PEPCK1 and FASN in hepatocytes

**Fig. 6:**
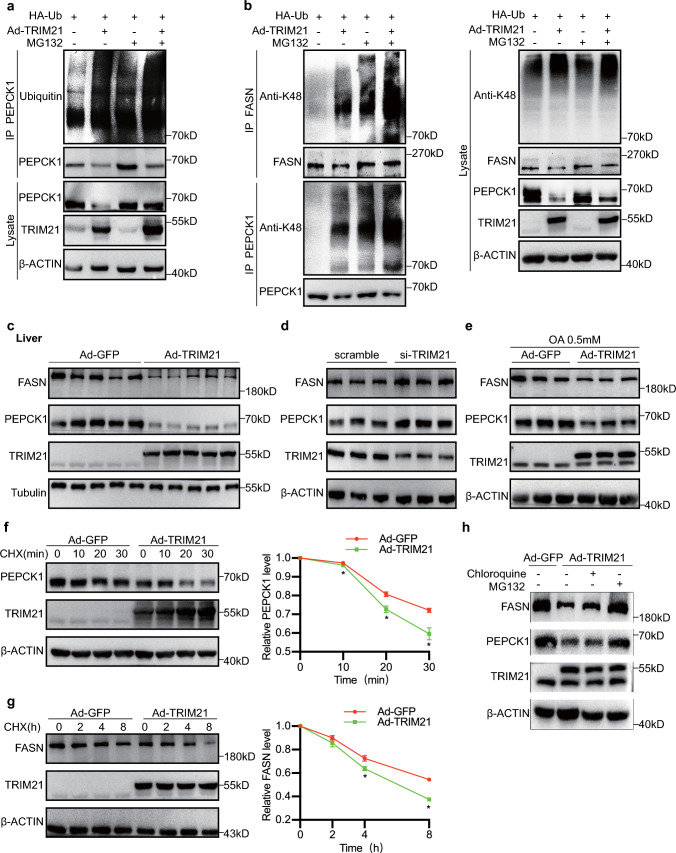
TRIM21 ubiquitinated and degraded PEPCK1 and FASN in hepatocytes. **a** Mouse primary hepatocytes were transfected with HA-ubiquitin (Ub) and Ad-TRIM21 for 48 h; 4 h after treatment with proteasome inhibitor MG132 (10 μmol/L), the cells were lysed, and the supernatant was collected for immunoprecipitation (IP) experiment. Ubiquitin antibody was used for western blot analysis to measure exogenous PEPCK1 ubiquitination. **b** K48 levels of PEPCK1 or FASN after TRIM21 overexpression and in response to palm acid treatment in mouse primary hepatocytes. **c** The protein levels of PEPCK1 and FASN were measured in the livers of *db/db* mice injected with Ad-GFP or Ad-TRIM21 by western blotting analysis. **d** The expression of PEPCK1 and FASN was detected in mouse primary hepatocytes transfected with scramble si-RNA or si-TRIM21. **e** The expression of PEPCK1 and FASN was detected in mouse primary hepatocytes infected with Ad-GFP or Ad-TRIM21 under oleic acid (0.5 mM) treatment. **f**, **g** A cycloheximide (CHX) chase was performed to establish the time course of PEPCK1 or FASN biogenesis. Mouse primary hepatocytes were infected with Ad-GFP or Ad-TRIM21 for 24 h; then the cells were with CHX (50 mmol/L) for 0, 2, 4, or 8 h. Western blot analysis was performed, and the relative FASN expression was calculated. The PEPCK1 expression was detected after coculture with CHX (50 mmol/L) for 0, 10, 20, or 30 min. Quantitation of western blot data for PEPCK1 and FASN proteins as in right panels. *P < 0.05 vs. Ad-GFP. *n* = 3. **h** Mouse primary hepatocytes were infected with Ad-GFP or Ad-TRIM21 for 48 h and then treated with MG132 (10 μmol/L) or chloroquine (50 μmol/L) for another 4 h; PEPCK1 or FASN expression was measured through western blot analysis. Data are presented as mean ± SEM

We also investigated whether TRIM21 mediated the ubiquitination of PEPCK1 in hepatocytes using ubiquitination assays. We transfected ubiquitin (Ub) plasmids into mouse primary hepatocytes with or without Ad-TRIM21 infection. PEPCK1 was immunoprecipitated and the protein expression of ubiquitin was observed by western blotting. Indeed, PEPCK1 was ubiquitylated, and the levels of ubiquitinated PEPCK1 were increased in TRIM21-overexpressing cells (Fig. 6a). Of note, TRIM21 RING^Δ^ failed to bind to PEPCK1 and FASN, indicating the RING-finger domain was needed to TRIM21 function in PEPCK1 and FASN ubiquitination. In addition, we sought a better understanding of ubiquitin-mediated PEPCK1 and FASN degradation by transfecting HA-tagged ubiquitin plasmids into mouse primary hepatocytes with or without Ad-TRIM21 infection. PEPCK1 and FASN were immunoprecipitated and the K48-linkage specific polyubiquitin was observed by western blotting. As shown by western blot analysis, in contrast to vehicle-treated cells, PEPCK1 and FASN were K48 polyubiquitinated in mouse primary hepatocytes treated with Ad-TRIM21 instead of K63 polyubiquitinated (Fig. 6b, Supplementary Fig. 3).

K48 ubiquitinated proteins are targeted for degradation by proteasomal or autophagy mechanisms. For these studies, we then investigated the effect of TRIM21 on the expression of PEPCK1 and FASN. Consistent with the ubiquitin level, an expression level test supported the reduction in PEPCK1 and FASN protein levels in mouse liver samples by forced expression of TRIM21 (Fig. 6c). We then used TRIM21 knockdown and TRIM21 overexpressing in mouse primary hepatocytes to further investigate the role of TRIM21. We knocked down TRIM21 expression in primary hepatocytes using siRNA and used scrambled siRNA as control (Supplementary Fig. 4). Notably, the expression of PEPCK1 and FASN were upregulated by TRIM21 knockdown but inhibited by TRIM21 overexpression in vitro (Fig. 6d, e). Furthermore, we explored the effect of TRIM21 on the stability of PEPCK1 or FASN expression by performing a cycloheximide (CHX, a protein translation inhibitor) chase assay. The half-life of PEPCK1 and FASN protein was reduced in mouse primary hepatocytes infected with Ad-TRIM21 under the treatment of palmitic acid (Fig. 6f, g). In addition, we monitored the expression levels of PEPCK1 and FASN treated with Ad-TRIM21 in the absence and presence of chloroquine or MG132 to inhibit autophagy or proteasome activity. As shown by western blot analysis (Fig. 6h), the degradation of PEPCK1 and FASN was suppressed entirely by MG132 but not chloroquine. These data suggested that TRIM21 inhibited PEPCK1 and FASN expression by the ubiquitin–proteasome pathway.

### TRIM21 suppressed hepatic glucose and lipid metabolic disorders through the regulation of PEPCK1 and FASN expression

**Fig. 7:**
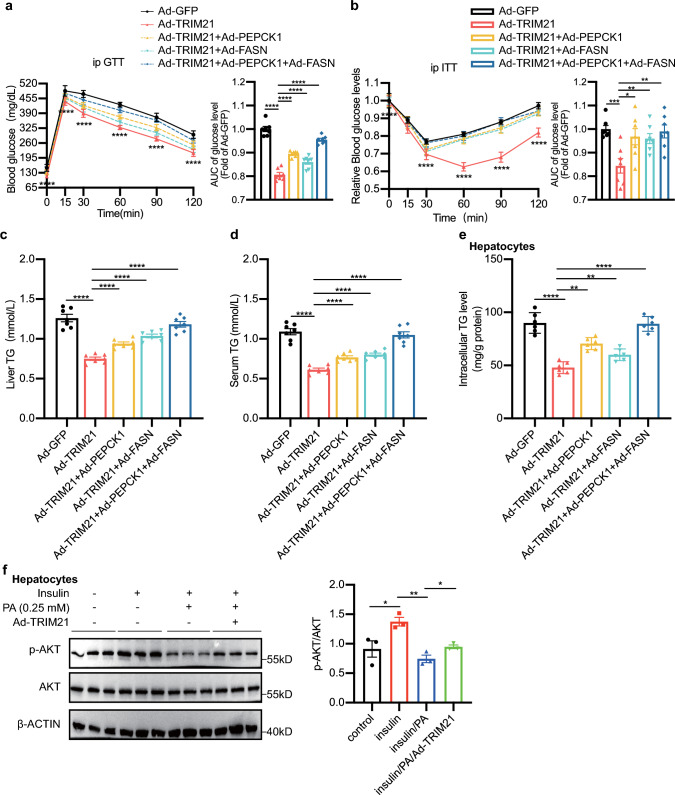
TRIM21 suppressed hepatic glucose and lipid metabolic disorders by regulating PEPCK1 and FASN expression. **a**–**d** C57BL/6J mice fed HFD for 12 weeks were injected with Ad-TRIM21, Ad-PEPCK1, and/or Ad-FASN via tail vein injection. Seven days after injection, GTT (**a**), ITT (**b**), liver TG (**c**), and serum TG (**d**) were measured as indicated. *P < 0.05, **P < 0.01, ***P < 0.001, ****P < 0.0001 vs. Ad-GFP group. *n* = 7 mice for each group. **e** Mouse primary hepatocytes were co-infected with Ad-TRIM21, Ad-PEPCK1, and/or Ad-FASN for 24 h, then treated with palmitic acid (0.25 mM) and oleic acid (0.5 mM) for another 24 h. Intracellular TG levels were measured. *P < 0.05, **P < 0.01, ***P < 0.001, ****P < 0.0001 vs. Ad-GFP group. *n* = 6 per group. **f** Mouse primary hepatocytes were infected with Ad-GFP or Ad-TRIM21 for 24 h before being treated with/without palmitic acid (0.25 mM) for 16 h. Before collection, the cells were incubated with 100 nmol/L insulin for 20 min to stimulate insulin signaling. β-Actin was used as the loading control. Quantification data was shown in the lower panel. Data are presented as mean ± SEM. *P < 0.05, **P < 0.01 vs. control group. *n* = 3 per group

Consistent with results of some other published studies [25], overexpression of hepatic PEPCK1 or FASN also considerably elevated blood glucose levels, liver TG and serum TG levels of HFD-fed mice in our current study (Supplementary Fig. 5). To investigate whether PEPCK1 and FASN were involved in the suppressive effect of TRIM21 on hepatic glucose and lipid metabolic disorders, we injected Ad-PEPCK1 and/or Ad-FASN into HFD-fed mice with TRIM21 overexpression. The protective effect of TRIM21 overexpression on HFD-induced glucose intolerance and insulin resistance was partially abolished by PEPCK1 or FASN overexpression (Fig. 7a, b, upper panels). Notably, the reversal effect was more significant by both PEPCK1 and FASN overexpression. The area under the curve (AUC) of the GTT and ITT in PEPCK1 or FASN overexpression mice was also increased (Fig. 7a, b, lower panels). PEPCK1 and/or FASN overexpression also partially reversed the suppressed hepatic TG levels and serum TG in the HFD-diet mice overexpressing TRIM21 (Fig. 7c, d).

Consistently, ectopic TRIM21 expression attenuated intracellular TG accumulation in mouse primary hepatocytes exposed to the combination of palmitic acid and oleic acid, which was reversed by PEPCK1 and/or FASN overexpression (Fig. 7e). In addition, TRIM21 overexpression led to an increase in insulin-stimulated phosphorylation of AKT in mouse primary hepatocytes when exposed to palmitic acid (Fig. 7f, quantified in Fig. 7f, right panel). These results indicated that TRIM21 suppressed hepatic glucose and lipid metabolic disorders through the regulation of PEPCK1 and FASN expression.

## Discussion

**Fig. 8:**
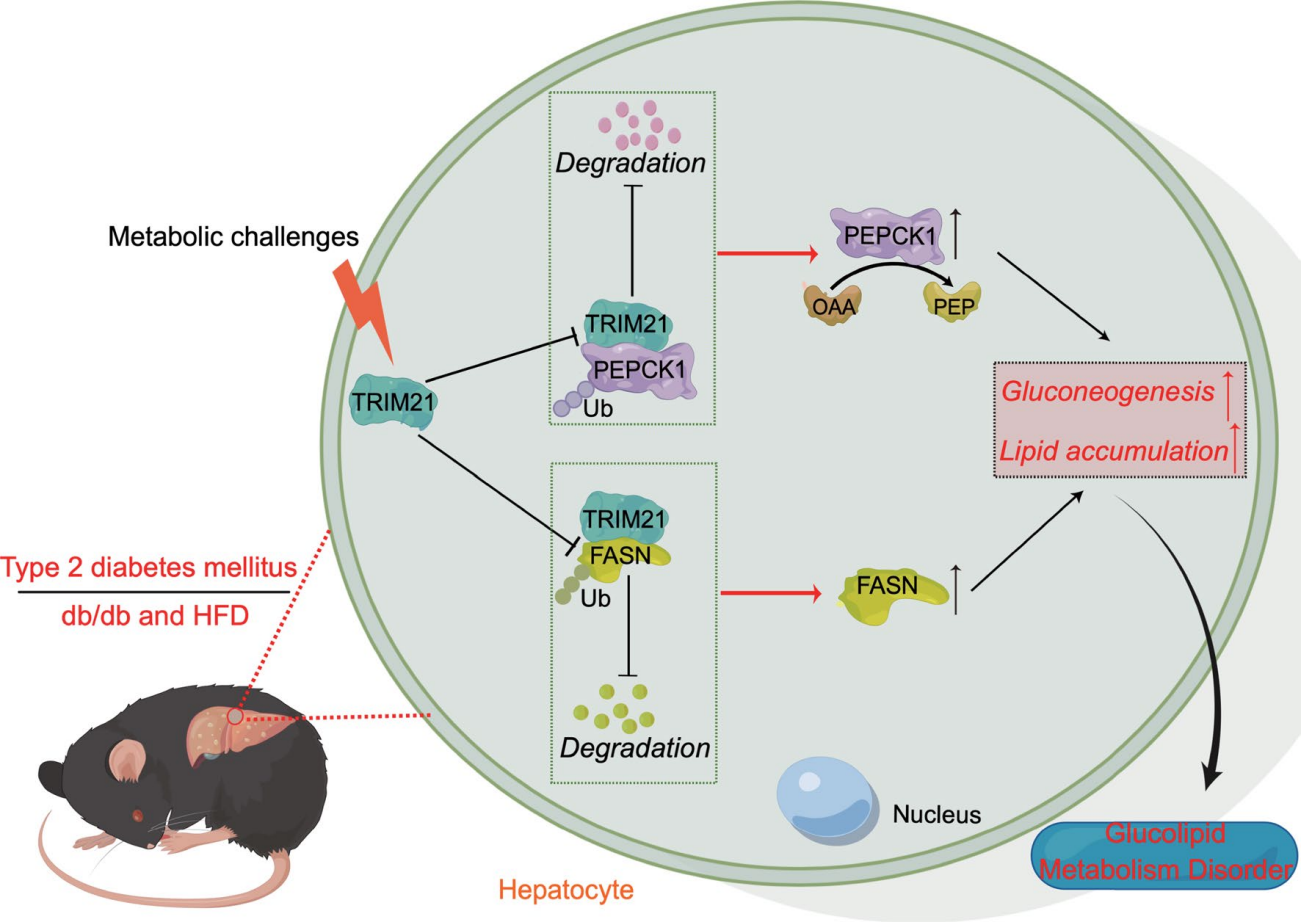
Graphical abstracts. The decreased TRIM21 expression negatively regulates PEPCK1 and FASN, leading to sustained gluconeogenesis and excessive lipid synthesis in hepatocytes, thereby greatly exacerbating insulin resistance and glucose intolerance in type 2 diabetes mellitus. *Ub* ubiquitin, *OAA* oxaloacetate, *PEP* phosphoenolpyruvate

Type 2 diabetes mellitus is a significant public health problem due to its high prevalence worldwide. Data from the studies from our group and others supported the concept that impaired regulation of glucose and lipid metabolism in the liver plays a pivotal role in the pathogenesis of T2DM. However, the regulatory mechanisms that regulate hepatic glycolipid metabolism disorder are still incompletely elucidated. In the current study, we identify E3 ligase TRIM21 as a novel player in hepatic glycolipid metabolism abnormalities that leads to the development and progression of type 2 diabetes mellitus. We propose a model in which the downregulated TRIM21 attenuated its suppression of PEPCK1 and FASN expression in the liver through protein degradation, which consequently leads to sustained gluconeogenesis and excessive lipid synthesis in hepatocytes, thereby greatly exacerbating insulin resistance and glucose intolerance (Fig. 8).

TRIM21 has been demonstrated as a protective molecule by elaborating E3 ligase activity. A previous study has shown that TRIM21 protects against intestinal mucosal inflammation by inhibiting TH1 and TH17 cell activation and differentiation [17]. In CD4^+^T cells, TRIM21 degraded multiple metabolic enzymes in a proteasome-dependent manner to alleviate the onset of T cell-mediated colitis [26]. Though the majority of the effect of TRIM21 thus far has focused on cancerous and infective diseases [16], our current study reported the essential role of hepatic TRIM21 in T2DM for the first time. Hepatic TRIM21 is markedly reduced in steatosis patients and obese diabetic mice.

Interestingly, the liver exhibited the strongest TRIM21 expression among the major metabolic organs, suggesting that the impacts of TRIM21 on metabolism could be more specific in the liver. We further identified that TRIM21 regulates glucose, lipid metabolism, and insulin signaling in the liver. Notably, hepatic TRIM21 overexpression in diet-induced and gene-induced diabetic mice attenuates hyperglycemia and hyperlipidemia. Similarly, overexpression of TRIM21 inhibits steatosis in vitro. These results strongly indicate that reduced TRIM21 expression is probably a cause, not a consequence, of obesity-associated metabolic disorder, which was further supported by the hepatic TRIM21 knockdown experiment in mice, which promoted liver steatosis and insulin resistance. However, there was no difference in body weight and food intake in the adenovirus-TRIM21 injection as well as in Ad-shTRIM21-injected mice. We consider the explanations lying in two aspects. Firstly, overexpression of TRIM21 improved hepatic insulin sensitivity and ameliorated systemic glucose and lipid metabolism disorders. Secondly, upregulation of TRIM21 expression can reduce triglyceride production in the liver and TG level in the circulation, thereby ameliorating the insulin resistance of other organs and systemic metabolic disorders. Since the food intake and body weight of mice were not affected, we speculated that TRIM21 overexpression caused significant reductions in liver triglycerides and serum triglycerides may redistribute to adipose tissue or is excreted in the feces.

To confirm the essential effect of TRIM21 on metabolism in an in vivo setting, we applied an adenoviral gene transfer technique to overexpress TRIM21 in the liver of mice. Although the intravenous application of adenoviral particles was not explicitly developed for liver transduction, most of the viral particles were retained in the liver, thus effectively transducing liver tissue [27]. Since the mice used to activate anti-viral immune response following intravenous adenovirus injection, we allowed them to recover for seven days before further experiments. Seven days after administering adenoviral particles through the tail vein injection, we still can obtain more than 85% transduction efficiency in the hepatocytes of db/db mice and HFD mice. Meanwhile, injection of TRIM21 adenoviral particles did not alternate TRIM21 expression in other major metabolic organs, such as adipose tissue, skeleton muscle, pancreas, etc., which was determined by Western blot (data not shown). Based on these findings, intravenous application of adenoviral particles was used here as a reliable in vivo model to study TRIM21's impacts on hepatic metabolism.

As E3 ubiquitin ligases, most TRIM family members are pivotal players in the posttranslational modification of specific proteins by promoting their degradation. Data obtained from the current study demonstrated that TRIM21 directly targets the key metabolic enzymes involved in gluconeogenesis (i.e., PEPCK1) and fatty acid synthesis (i.e., FASN) in the liver. The RING domain of TRIM21 is mainly responsible for its interaction with and ubiquitination of PEPCK1 or FASN. PEPCK1 is a critical enzyme that catalyzes the first committed and rate-limiting step of gluconeogenesis [28]. Diabetic mice (*db/db*) with loss of hepatic PEPCK1 have an improved ability to regulate glycemia, insulin sensitivity, and dyslipidemia [29]. Due to the critical role of elevated gluconeogenesis in the development of type 2 diabetes mellitus, mechanisms involved in PEPCK1 regulation have been extensively investigated [30–32]. Among these, post-transcriptional modulation, especially ubiquitination, has been shown to rapidly regulate PEPCK1 activity and expression [33, 34]. In this study, we identified the ubiquitination-mediated degradation of PEPCK1 by TRIM21. We also validated that TRIM21 catalyzed K48-linked ubiquitination of PEPCK1. Furthermore, PEPCK1 overexpression partially abolished the protective effect of TRIM21 overexpression on HFD-induced hepatosteatosis, insulin resistance, and glucose intolerance, consistent with PEPCK1 function in gluconeogenesis. Besides our findings, it has been reported that PEPCK also can be degredated by the E3 ligase UBR5 through proteasome system. This process was regulated by glucose concentration-dependent PEPCK1 acetylation [30].

Numerous studies have revealed the critical involvement of hepatosteatosis in hepatic insulin resistance and glucose intolerance [35, 36]. Increased hepatic fat accumulation is associated with decreased insulin clearance and sensitivity in type 2 diabetes mellitus patients [37, 38]. TRIM21 has been shown to directly mediate lipid metabolism in controlling FASN ubiquitination and degradation in tumors [20, 21]. In the present study, we demonstrated that FASN was degraded by TRIM21 in hepatocytes. Moreover, TRIM21 mediated alleviation of hepatic lipid deposition and improvement of insulin resistance and glucose intolerance was partially abolished in the presence of FASN. Since hepatic steatosis has been regarded as a driving force for insulin resistance and type 2 diabetes mellitus [4], the improved insulin sensitivity by TRIM21 could ameliorate hepatic glucose and lipid metabolic disorders by ubiquitination of FASN expression. While it has been shown in previous clinical trials that FASN inhibitors can reduce hepatic de novo lipogenesis and steatosis in patients with obesity and NAFLD [39, 40], it cannot be ruled out that the improvement in steatosis resulting from the suppression of FASN expression by TRIM21 may have also been influenced by fatty acid re-esterification. As far as the liver was specifically concerned, neither key regulator of the insulin signaling pathway was identified as the substrate of TRIM21 by pull-down mass spectrometry. However, TRIM21 significantly improved insulin sensitivity and enhanced the activation of the insulin pathway, which was represented by upregulated Akt phosphorylation in the livers of obese diabetic mice. Here are our considerations for this issue. Firstly, TRIM21 may affect the insulin signaling pathway independent of its E3 ligase activity. Secondly, there are supposed that one or more potential TRIM21 substrate(s) may serve as the regulator(s) of the insulin signaling pathway but cannot be identified by coimmunoprecipitation or mass spectrometry techniques due to its relatively low abundance. We must use more sensitive methods to screen out the TRIM21 partner molecules to elucidate their effects on insulin sensitivity in the liver in the future. Nonetheless, our result still indicated that targeting TRIM21 might be a promising strategy for improving insulin sensitivity in the liver. It has been reported that PEPCK1 and FASN have opposite effects on hepatic metabolism in healthy individuals [41, 42]. However, the expression of TRIM21, FASN, and PEPCK1 did not show significant association in the liver of wild-type mice (data not shown), implying that TRIM21 may have distinct roles in hepatic metabolism under physiological and pathological conditions.

We noticed that the role of TRIM21 in hepatic cell carcinoma pathogenesis still remains elusive and controversial. However, in our current study, hepatic ectopic expression significantly improves steatosis through degradation of FASN. This leads us to speculate that TRIM21 may play a crucial role in alleviating hepatic cell carcinoma development caused by metabolic disorders.

In summary, this study demonstrates a novel role of hepatic TRIM21 in glucose, lipid metabolism, and insulin signaling through the regulation of PEPCK1 and FASN. Negative regulation of PEPCK1 and FASN by TRIM21 reduces sustained gluconeogenesis and inhibits steatosis in hepatocytes, thereby significantly alleviating insulin resistance and glucose intolerance. These findings suggest that TRIM21 may be a promising novel target for treating type 2 diabetes mellitus.

## Conclusions

The results of this study support the concept that TRIM21 improves hepatic glucose and lipid metabolic disorders through its binding to PEPCK and FASN. Moreover, it is worthwhile to elucidate the mechanism of how TRIM21 expression was regulated in the liver with metabolic disorders in the future.

### Supplementary Information

Below is the link to the electronic supplementary material.

Supplementary file1 (DOCX 813 KB)

## Data Availability

The datasets, adenoviruses, and plasmids generated and/or analyzed during the current study are available from the corresponding authors upon reasonable request.

## Abbreviations

TRIM21: Tripartite motif-containing protein 21
FASN: Fatty acid synthase
PEPCK1: Phosphoenolpyruvate carboxykinase-1
HFD: High-fat-diet
NAFLD: Nonalcoholic fatty liver disease
NASH: Nonalcoholic steatohepatitis
PA: Palmitic acid
OA: Oleic acid
Dex: Dexamethasone
GTT: Glucose tolerance test
PTT: Pyruvate tolerance test
ITT: Insulin tolerance test
H&E: Hematoxylin and eosin
TG: Triglyceride
TC: Total cholesterol
ALT: Alanine aminotransferase
AST: Aspartate aminotransferase
CHX: Cycloheximide
