# Supplementary material for: TRIM21 ameliorates hepatic glucose and lipid metabolic disorders in type 2 diabetes mellitus by ubiquitination of PEPCK1 and FASN
Source: Cell Mol Life Sci. 2023 May 30;80(6):168. doi: 10.1007/s00018-023-04820-w (PMC10229743; doi:10.1007/s00018-023-04820-w)
Supplement: Supplementary file 1 — Supplementary file1 (DOCX 813 KB) [file 18_2023_4820_MOESM1_ESM.docx]

**Supplementary data**

**Journal name:** Cellular and Molecular Life Sciences

**Title:** TRIM21 ameliorates hepatic glucose and lipid metabolic disorders in type 2 diabetes mellitus by ubiquitination of PEPCK1 and FASN

**Authors:**

Kaini Zhang^1#^, Chen Yang^2#^, Xin Zhou^1#^, Jin Liang^3^, Jianjin Guo^4, 5^, Min Li^2^, Yi Zhang^6^, Shulin Shao^7^, Peng Sun^3^, Kai Li^3^, Jingjing Huang^8^, Fang Chen^3*^, Xiubin Liang^1*^, Dongming Su^2*^

1. Department of Pathophysiology, Nanjing Medical University, Nanjing 211166, China

2. Department of Pathology, Nanjing Medical University, Nanjing 211166, China

3. Key Laboratory of Human Functional Genomics of Jiangsu Province, Nanjing Medical University, Nanjing 211166, China

4. Department of General Medicine, Shanxi Bethune Hospital, Shanxi Academy of Medical Sciences, Tongji Shanxi Hospital, Third Hospital of Shanxi Medical University, Taiyuan 030032, China

5. Department of General Medicine, Tongji Hospital, Tongji Medical College, Huazhong University of Science and Technology, Wuhan 430030, China

6. Department of Pathology, Jiangsu Cancer Hospital, Jiangsu Institute of Cancer Research, Nanjing Medical University Affiliated Cancer Hospital, Nanjing 211800, China

7. Department of Laboratory, Nanjing Pukou Hospital of Traditional Chinese Medicine, Nanjing 211800, China

8. Department of Geriatrics, the Fourth Affiliated Hospital of Nanjing Medical University, Nanjing 211166, China

# Kaini Zhang, Chen Yang and Xin Zhou contributed equally to this work.

Contact Information:

*Corresponding authors: Dongming Su (sudongming@njmu.edu.cn), Xiubin Liang (liangxiubin@njmu.edu.cn), or Fang Chen (chenfang@njmu.edu.cn). Tel: +86-025- 87115706; Fax: +86-025-87115706

**Figure S1. Body weight, food intake and insulin secretion were not affected by** **Adenovirus TRIM21 (Ad-TRIM21). Consistently, body weight and food intake were not affected by Adenovirus shTRIM21 (Ad-shTRIM21) injection.**

(**a, d**) Body weight. (**b, e**) Food intake. (**c**) Insulin secretion. ns. not significant. n = 5(**a**-**c**) or n = 7 (**d**, **e**) for each group.

**Figure S2. Expression levels of key regulators of lipid metabolism, classified as adipogenesis, β-oxidation, and uptake.**

Real-time qPCR analysis showed hepatic TRIM21 repressed the expression levels of several lipogenic genes, including fatty acid synthase (Fasn) and stearoyl-CoA desaturase 1 (Scd1), while several β-oxidation and fatty acid uptake genes were unchanged. Data were presented as mean ± SEM. **P* < 0.05, ***P* < 0.01, ****P* < 0.001, *****P* < 0.0001 vs. Ad-GFP group. n = 3 for each group.

**Figure S3.** **PEPCK1 and FASN were not K63-linkage polyubiquitinated in mouse primary hepatocytes treated with or without Ad-TRIM21.**

K63 levels of PEPCK1 or FASN after TRIM21 overexpression and in response to palm acid treatment in mouse primary hepatocytes.

**Figure S4.** **The knock-down efficiency of si-TRIM21 was more than 50% in the mouse primary hepatocytes.**

The mouse primary hepatocytes were transfected with si-TRIM21 (#148, #1056, #411) to knock down TRIM21 expression, scrambled si-RNA as control. Western blot assays showed the knock-down efficiency of si-TRIM21 (left). Quantitative measurement of TRIM21 protein relative to tubulin (right). Data were presented as mean ± SEM. ***P* < 0.01 vs. scrambled group. n = 3 for each group.

**Figure S5.** **Hepatic PEPCK1 and FASN expression were increased in HFD mice. Overexpression of hepatic PEPCK1 or FASN considerably elevated blood glucose levels, liver TG and serum TG levels in HFD-fed mice.**

(**a**)The protein levels of PEPCK1 and FASN were measured in the livers of male HFD-fed mice by western blotting analysis. The comparisons are made with respective age-matched and sex-matched controls. Quantification data was shown in the right panel. Data are presented as mean ± SEM. **P* < 0.05, ***P* < 0.01 vs. ND. The data are normalized to tubulin (n =5 for all groups). (**b**-**d**) C57BL/6J mice fed HFD for 12 weeks were injected with Ad-GFP, Ad-PEPCK1, or Ad-FASN via tail vein injection. Blood glucose levels (**b**), liver TG levels (**c**), and serum TG levels (**d**) were measured as indicated. **P* < 0.05 vs. Ad-GFP group. n = 7 mice for each group.

**Table 1. List of the interactive proteins of TRIM21 in Ad-TRIM21-transfected cells analyzed by mass spectrometry.**

| **First protein** | **Gene name** | **Description** |
| --- | --- | --- |
| P15924 | DSP | Desmoplakin |
| Q14C86-2 | GAPVD1 | Isoform 2 of GTPase-activating protein and VPS9 domain-containing protein 1 |
| P19474 | TRIM21 | E3 ubiquitin-protein ligase TRIM21 |
| P23458 | JAK1 | Tyrosine-protein kinase JAK1 |
| A0A0A0MRF6 | AKAP9 | A-kinase anchor protein 9 |
| P11021 | HSPA5 | 78 kDa glucose-regulated protein |
| Q9NSD9 | FARSB | Phenylalanine--tRNA ligase beta subunit |
| P11142 | HSPA8 | Heat shock cognate 71 kDa protein |
| P10809 | HSPD1 | 60 kDa heat shock protein, mitochondrial |
| Q9UPN9-2 | TRIM33 | Isoform Beta of E3 ubiquitin-protein ligase TRIM33 |
| P02768 | ALB | Serum albumin |
| P38646 | HSPA9 | Stress-70 protein, mitochondrial |
| P14923 | JUP | Junction plakoglobin |
| P60709 | ACTB | Actin, cytoplasmic 1 |
| P63261 | ACTG1 | Actin, cytoplasmic 2 |
| P0DMV9 | HSPA1B | Heat shock 70 kDa protein 1B |
| P13861-2 | PRKAR2A | Isoform 2 of cAMP-dependent protein kinase type II-alpha regulatory subunit |
| Q5JP53 | TUBB | Tubulin beta chain |
| O75592-2 | MYCBP2 | Isoform 2 of E3 ubiquitin-protein ligase MYCBP2 |
| E7EQ69 | NAA50 | N-alpha-acetyltransferase 50 |
| P29508 | SERPINB3 | Serpin B3 |
| F5H5D3 | TUBA1C | Tubulin alpha chain |
| P68371 | TUBB4B | Tubulin beta-4B chain |
| P48594 | SERPINB4 | Serpin B4 |
| Q5VTE0 | EEF1A1P5 | Putative elongation factor 1-alpha-like 3 |
| P51659 | HSD17B4 | Peroxisomal multifunctional enzyme type 2 |
| P08238 | HSP90AB1 | Heat shock protein HSP 90-beta |
| Q3BDU5 | LMNA | Prelamin-A/C |
| Q8IYB1 | MB21D2 | Protein MB21D2 |
| P11177-2 | PDHB | Isoform 2 of Pyruvate dehydrogenase E1 component subunit beta, mitochondrial |
| P68133 | ACTA1 | Actin, alpha skeletal muscle |
| Q06830 | PRDX1 | Peroxiredoxin-1 |
| P23396 | RPS3 | 40S ribosomal protein S3 |
| Q13268 | DHRS2 | Dehydrogenase/reductase SDR family member 2, mitochondrial |
| P06733 | ENO1 | Alpha-enolase |
| P78347-2 | GTF2I | Isoform 2 of General transcription factor II-I |
| P07900 | HSP90AA1 | Heat shock protein HSP 90-alpha |
| P19338 | NCL | Nucleolin |
| P05109 | S100A8 | Protein S100-A8 |
| F8VPD4 | CAD | CAD protein |
| Q9H8M5-2 | CNNM2 | Isoform 2 of Metal transporter CNNM2 |
| F6S8N6 | PCMT1 | Protein-L-isoaspartate(D-aspartate) O-methyltransferase |
| P62269 | RPS18 | 40S ribosomal protein S18 |
| P31151 | S100A7 | Protein S100-A7 |
| P05141 | SLC25A5 | ADP/ATP translocase 2 |
| Q04828 | AKR1C1 | Aldo-keto reductase family 1 member C1 |
| A0A140TA49 | C4A | Complement C4-A |
| Q02413 | DSG1 | Desmoglein-1 |
| P17066 | HSPA6 | Heat shock 70 kDa protein 6 |
| A6NNN6 | PCM1 | Pericentriolar material 1 protein |
| P63244 | RACK1 | Receptor of activated protein C kinase 1 |
| P62424 | RPL7A | 60S ribosomal protein L7a |
| P39019 | RPS19 | 40S ribosomal protein S19 |
| P06702 | S100A9 | Protein S100-A9 |
| Q562R1 | ACTBL2 | Beta-actin-like protein 2 |
| Q5D862 | FLG2 | Filaggrin-2 |
| P04406 | GAPDH | Glyceraldehyde-3-phosphate dehydrogenase |
| P62805 | HIST1H4A | Histone H4 |
| P61313 | RPL15 | 60S ribosomal protein L15 |
| P39023 | RPL3 | 60S ribosomal protein L3 |
| A8MUD9 | RPL7 | 60S ribosomal protein L7 |
| M0R0F0 | RPS5 | 40S ribosomal protein S5 (Fragment) |
| P22532 | SPRR2D | Small proline-rich protein 2D |
| P22531 | SPRR2E | Small proline-rich protein 2E |
| J3QS39 | UBB | Polyubiquitin-B (Fragment) |
| O60701-2 | UGDH | Isoform 2 of UDP-glucose 6-dehydrogenase |
| H3BQN4 | ALDOA | Fructose-bisphosphate aldolase |
| A0A0U1RQF0 | FASN | Fatty acid synthase |
| P20930 | FLG | Filaggrin |
| E9PCY7 | HNRNPH1 | Heterogeneous nuclear ribonucleoprotein H |
| Q00839-2 | HNRNPU | Isoform Short of Heterogeneous nuclear ribonucleoprotein U |
| Q9NZI8 | IGF2BP1 | Insulin-like growth factor 2 mRNA-binding protein 1 |
| P00338-4 | LDHA | Isoform 4 of L-lactate dehydrogenase A chain |
| P07237 | P4HB | Protein disulfide-isomerase |
| Q8WVV4 | POF1B | Protein POF1B |
| P62937 | PPIA | Peptidyl-prolyl cis-trans isomerase A |
| F5H018 | RAN | GTP-binding nuclear protein Ran (Fragment) |
| P36578 | RPL4 | 60S ribosomal protein L4 |
| H0YEN5 | RPS2 | 40S ribosomal protein S2 (Fragment) |
| P62701 | RPS4X | 40S ribosomal protein S4, X isoform |
| Q5JR95 | RPS8 | 40S ribosomal protein S8 |
| Q5T750 | XP32 | Skin-specific protein 32 |
| H9KV75 | ACTN1 | Alpha-actinin-1 |
| P02765 | AHSG | Alpha-2-HS-glycoprotein |
| P25705-2 | ATP5A1 | Isoform 2 of ATP synthase subunit alpha, mitochondrial |
| H0YH81 | ATP5B | ATP synthase subunit beta (Fragment) |
| P01024 | C3 | Complement C3 |
| P48729 | CSNK1A1 | Casein kinase I isoform alpha |
| P49674 | CSNK1E | Casein kinase I isoform epsilon |
| A0A1B0GW44 | CTSD | Cathepsin D |
| P81605 | DCD | Dermcidin |
| P0C2W1 | FBXO45 | F-box/SPRY domain-containing protein 1 |
| A0A0U1RR32 | hCG_2039566 | Histone H2A |
| U3KQK0 | HIST1H2BN | Histone H2B |
| P04196 | HRG | Histidine-rich glycoprotein |
| Q86YZ3 | HRNR | Hornerin |
| Q15365 | PCBP1 | Poly(rC)-binding protein 1 |
| Q15084-3 | PDIA6 | Isoform 3 of Protein disulfide-isomerase A6 |
| O43175 | PHGDH | D-3-phosphoglycerate dehydrogenase |
| P12273 | PIP | Prolactin-inducible protein |
| P62913 | RPL11 | 60S ribosomal protein L11 |
| P62829 | RPL23 | 60S ribosomal protein L23 |
| H0YLP6 | RPL28 | 60S ribosomal protein L28 |
| D3YTB1 | RPL32 | 60S ribosomal protein L32 (Fragment) |
| M0QZC5 | RPS11 | 40S ribosomal protein S11 |
| E9PKH2 | SERPINH1 | Serpin H1 |
| A8MXB7 | SNX24 | Sorting nexin-24 |
| E7EX29 | YWHAZ | 14-3-3 protein zeta/delta (Fragment) |
| O95831-3 | AIFM1 | Isoform 3 of Apoptosis-inducing factor 1, mitochondrial |
| H0YKZ7 | ANXA2 | Annexin (Fragment) |
| P05089 | ARG1 | Arginase-1 |
| E9PP50 | CFL1 | Cofilin-1 (Fragment) |
| A0A087WVQ6 | CLTC | Clathrin heavy chain |
| P14868-2 | DARS | Isoform 2 of Aspartate--tRNA ligase, cytoplasmic |
| J3KTA4 | DDX5 | Probable ATP-dependent RNA helicase DDX5 |
| F8WCJ1 | EIF5A2 | Eukaryotic translation initiation factor 5A |
| P07305 | H1F0 | Histone H1.0 |
| K7EMV3 | H3F3B | Histone H3 |
| F8W6P5 | HBB | Hemoglobin subunit beta (Fragment) |
| P69892 | HBG2 | Hemoglobin subunit gamma-2 |
| P16403 | HIST1H1C | Histone H1.2 |
| Q96A08 | HIST1H2BA | Histone H2B type 1-A |
| F8W6I7 | HNRNPA1 | Heterogeneous nuclear ribonucleoprotein A1 |
| P61604 | HSPE1 | 10 kDa heat shock protein, mitochondrial |
| O75874 | IDH1 | Isocitrate dehydrogenase [NADP] cytoplasmic |
| A0A0A0MS07 | IGHG1 | Ig gamma-1 chain C region (Fragment) |
| P33176 | KIF5B | Kinesin-1 heavy chain |
| P47929 | LGALS7 | Galectin-7 |
| Q6ZVX7 | NCCRP1 | F-box only protein 50 |
| E5RGH3 | PABPC1 | Polyadenylate-binding protein 1 (Fragment) |
| E9PL24 | PDE4DIP | Myomegalin |
| O00151 | PDLIM1 | PDZ and LIM domain protein 1 |
| H3BT25 | PKM | Pyruvate kinase PKM (Fragment) |
| P27169 | PON1 | Serum paraoxonase/arylesterase 1 |
| P23284 | PPIB | Peptidyl-prolyl cis-trans isomerase B |
| P32119 | PRDX2 | Peroxiredoxin-2 |
| P27694 | RPA1 | Replication protein A 70 kDa DNA-binding subunit |
| P62906 | RPL10A | 60S ribosomal protein L10a |
| P30050 | RPL12 | 60S ribosomal protein L12 |
| P26373 | RPL13 | 60S ribosomal protein L13 |
| P40429 | RPL13A | 60S ribosomal protein L13a |
| A0A087WXM6 | RPL17 | 60S ribosomal protein L17 (Fragment) |
| F8VUA6 | RPL18 | 60S ribosomal protein L18 (Fragment) |
| C9JD32 | RPL23 | 60S ribosomal protein L23 (Fragment) |
| P62750 | RPL23A | 60S ribosomal protein L23a |
| C9JXB8 | RPL24 | 60S ribosomal protein L24 |
| P47914 | RPL29 | 60S ribosomal protein L29 |
| H7C2W9 | RPL31 | 60S ribosomal protein L31 (Fragment) |
| P49207 | RPL34 | 60S ribosomal protein L34 |
| F8VZ45 | RPL6 | 60S ribosomal protein L6 (Fragment) |
| E9PKZ0 | RPL8 | 60S ribosomal protein L8 (Fragment) |
| D6RAN4 | RPL9 | 60S ribosomal protein L9 (Fragment) |
| F8VPE8 | RPLP0 | 60S acidic ribosomal protein P0 (Fragment) |
| F6U211 | RPS10 | 40S ribosomal protein S10 |
| P62263 | RPS14 | 40S ribosomal protein S14 |
| I3L3P7 | RPS15A | 40S ribosomal protein S15a |
| P60866 | RPS20 | 40S ribosomal protein S20 |
| P62851 | RPS25 | 40S ribosomal protein S25 |
| P62273 | RPS29 | 40S ribosomal protein S29 |
| D6R9B6 | RPS3A | 40S ribosomal protein S3a |
| P31947-2 | SFN | Isoform 2 of 14-3-3 protein sigma |
| P22528 | SPRR1B | Cornifin-B |
| P60174-4 | TPI1 | Isoform 4 of Triosephosphate isomerase |
| P49411 | TUFM | Elongation factor Tu, mitochondrial |
| Q8NBS9-2 | TXNDC5 | Isoform 2 of Thioredoxin domain-containing protein 5 |
| P13010 | XRCC5 | X-ray repair cross-complementing protein 5 |
| P27348 | YWHAQ | 14-3-3 protein theta |
| B1AMS2 | SEPT6 | Septin 6, isoform CRA_b |
| Q5T3U5-2 | ABCC10 | Isoform 2 of Multidrug resistance-associated protein 7 |
| D6R9I9 | ABCE1 | ATP-binding cassette sub-family E member 1 |
| E5RJR7 | ADAMTS1 | A disintegrin and metalloproteinase with thrombospondin motifs 1 (Fragment) |
| Q9P2N4-2 | ADAMTS9 | Isoform 3 of A disintegrin and metalloproteinase with thrombospondin motifs 9 |
| C9J299 | ADD2 | Beta-adducin (Fragment) |
| Q8IZF6-2 | ADGRG4 | Isoform 2 of Adhesion G-protein coupled receptor G4 |
| Q96MI9-2 | AGBL1 | Isoform 2 of Cytosolic carboxypeptidase 4 |
| H0YC22 | AGPAT5 | 1-acyl-sn-glycerol-3-phosphate acyltransferase epsilon (Fragment) |
| Q09666 | AHNAK | Neuroblast differentiation-associated protein AHNAK |
| A0A0J9YWL0 | AIM1 | Absent in melanoma 1 protein |
| Q9UKB5 | AJAP1 | Adherens junction-associated protein 1 |
| Q7Z591-5 | AKNA | Isoform 5 of AT-hook-containing transcription factor |
| F5H4B6 | ALDH16A1 | Aldehyde dehydrogenase family 16 member A1 |
| Q5SYQ7 | ALDH1A1 | Retinal dehydrogenase 1 (Fragment) |
| F5GZZ0 | ALKBH2 | DNA oxidative demethylase ALKBH2 (Fragment) |
| E9PHT9 | ANXA5 | Annexin |
| C9JPM4 | ARF4 | ADP-ribosylation factor 4 (Fragment) |
| A0A096LNY0 | ARHGEF9 | Rho guanine nucleotide exchange factor 9 (Fragment) |
| Q96EG1 | ARSG | Arylsulfatase G |
| H7C2G2 | ART4 | NAD(P)(+)--arginine ADP-ribosyltransferase (Fragment) |
| Q9NVI7-2 | ATAD3A | Isoform 2 of ATPase family AAA domain-containing protein 3A |
| O94823 | ATP10B | Probable phospholipid-transporting ATPase VB |
| Q9HD20-3 | ATP13A1 | Isoform C of Manganese-transporting ATPase 13A1 |
| K7ESE8 | BLMH | Bleomycin hydrolase (Fragment) |
| J3QQQ8 | BPTF | Nucleosome-remodeling factor subunit BPTF (Fragment) |
| A0A087WWL5 | C19orf68 | Uncharacterized protein C19orf68 |
| Q08AI8-3 | C2orf54 | Isoform 3 of Uncharacterized protein C2orf54 |
| A0A087WXS6 | C2orf74 | Uncharacterized protein C2orf74 (Fragment) |
| F8W9Z1 | CACNA1E | Voltage-dependent R-type calcium channel subunit alpha |
| K7EJB9 | CALR | Calreticulin (Fragment) |
| E9PLA9 | CAPRIN1 | Caprin-1 (Fragment) |
| C9JUG7 | CAPZA2 | F-actin-capping protein subunit alpha-2 |
| B1AK85 | CAPZB | F-actin-capping protein subunit beta |
| P31944 | CASP14 | Caspase-14 |
| K7ELA4 | CBX1 | Chromobox protein homolog 1 |
| Q9P1Z9-4 | CCDC180 | Isoform 4 of Coiled-coil domain-containing protein 180 |
| D6RDI7 | CCHCR1 | Coiled-coil alpha-helical rod protein 1 (Fragment) |
| F8VQ14 | CCT2 | T-complex protein 1 subunit beta |
| Q5SZX9 | CCT3 | T-complex protein 1 subunit gamma (Fragment) |
| P50991-2 | CCT4 | Isoform 2 of T-complex protein 1 subunit delta |
| A0A087WZ34 | CEP250 | Centrosome-associated protein CEP250 (Fragment) |
| Q5T0N1-6 | CFAP70 | Isoform 4 of Cilia- and flagella-associated protein 70 |
| M0R1A8 | CFLAR | CASP8 and FADD-like apoptosis regulator (Fragment) |
| F8W6H2 | CHPF | Chondroitin sulfate synthase 2 |
| J3QL22 | COPS3 | COP9 signalosome complex subunit 3 |
| B8ZZV9 | COQ10B | Coenzyme Q-binding protein COQ10 homolog B, mitochondrial |
| Q8N436 | CPXM2 | Inactive carboxypeptidase-like protein X2 |
| F8W642 | CS | Citrate synthase, mitochondrial (Fragment) |
| A0A0D9SFB3 | DDX3X | ATP-dependent RNA helicase DDX3X |
| G3V158 | DERA | 2-deoxyribose-5-phosphate aldolase homolog (C. elegans), isoform CRA_a |
| Q13574-3 | DGKZ | Isoform 3 of Diacylglycerol kinase zeta |
| Q658J9 | DKFZp434L0312 | Histone deacetylase 11 |
| Q86SW4 | DLST | Dihydrolipoamide acetyltransferase component of pyruvate dehydrogenase complex (Fragment) |
| A0A087WV90 | DMD | Dystrophin |
| M0R128 | DNAJB1 | DnaJ homolog subfamily B member 1 (Fragment) |
| A2A370 | DOCK8 | Dedicator of cytokinesis protein 8 |
| Q8IY82-2 | DRC7 | Isoform 2 of Dynein regulatory complex subunit 7 |
| Q08554-2 | DSC1 | Isoform 1B of Desmocollin-1 |
| E9PNW6 | EEF1D | Elongation factor 1-delta (Fragment) |
| D6RBD7 | EEF1E1 | Eukaryotic translation elongation factor 1 epsilon-1 |
| A4FU69-2 | EFCAB5 | Isoform 2 of EF-hand calcium-binding domain-containing protein 5 |
| E7EQG2 | EIF4A2 | Eukaryotic initiation factor 4A-II |
| F5H799 | EPSTI1 | Epithelial-stromal interaction protein 1 (Fragment) |
| Q03468-2 | ERCC6 | Isoform 2 of DNA excision repair protein ERCC-6 |
| P61571 | ERVK-21 | Endogenous retrovirus group K member 21 Rec protein |
| Q9NY74 | ETAA1 | Ewing's tumor-associated antigen 1 |
| Q6NZ36-2 | FAAP20 | Isoform 2 of Fanconi anemia core complex-associated protein 20 |
| Q01469 | FABP5 | Fatty acid-binding protein, epidermal |
| K7EK06 | FARSA | Phenylalanine--tRNA ligase alpha subunit (Fragment) |
| H3BTH6 | FBXO22 | F-box only protein 22 (Fragment) |
| A0A087WUA0 | FGA | Fibrinogen alpha chain |
| P02751-12 | FN1 | Isoform 12 of Fibronectin |
| G3V5N9 | FOS | Proto-oncogene c-Fos (Fragment) |
| H3BPE7 | FUS | RNA-binding protein FUS |
| Q13283 | G3BP1 | Ras GTPase-activating protein-binding protein 1 |
| Q9H3Q3 | GAL3ST2 | Galactose-3-O-sulfotransferase 2 |
| P50395-2 | GDI2 | Isoform 2 of Rab GDP dissociation inhibitor beta |
| O94808 | GFPT2 | Glutamine--fructose-6-phosphate aminotransferase [isomerizing] 2 |
| H3BRD8 | GLG1 | Golgi apparatus protein 1 (Fragment) |
| P00367-3 | GLUD1 | Isoform 3 of Glutamate dehydrogenase 1, mitochondrial |
| P15104 | GLUL | Glutamine synthetase |
| P17900 | GM2A | Ganglioside GM2 activator |
| Q53EU6 | GPAT3 | Glycerol-3-phosphate acyltransferase 3 |
| A0A087WU38 | GRIP2 | Glutamate receptor-interacting protein 2 |
| Q13255 | GRM1 | Metabotropic glutamate receptor 1 |
| K7EMR1 | GRN | Granulins (Fragment) |
| J3KRG2 | GSDMA | Gasdermin-A (Fragment) |
| Q92522 | H1FX | Histone H1x |
| Q5TGJ6 | HDGFL1 | Hepatoma-derived growth factor-like protein 1 |
| H0Y394 | HDLBP | Vigilin (Fragment) |
| P22626-2 | HNRNPA2B1 | Isoform A2 of Heterogeneous nuclear ribonucleoproteins A2/B1 |
| M0R0Y6 | HNRNPM | Heterogeneous nuclear ribonucleoprotein M |
| Q96ED9-2 | HOOK2 | Isoform 2 of Protein Hook homolog 2 |
| F8WE04 | HSPB1 | Heat shock protein beta-1 |
| P01594 | IGKV1-33 | Immunoglobulin kappa variable 1-33 |
| P01602 | IGKV1-5 | Immunoglobulin kappa variable 1-5 |
| A0A0C4DH67 | IGKV1-8 | Immunoglobulin kappa variable 1-8 |
| A0A0B4J2D9 | IGKV1D-13 | Immunoglobulin kappa variable 1D-13 |
| A0A0C4DH90 | IGKV3OR2-268 | Protein IGKV3OR2-268 (Fragment) |
| A0A075B6K4 | IGLV3-10 | Immunoglobulin lambda variable 3-10 |
| A0A075B6K5 | IGLV3-9 | HCG2043239 (Fragment) |
| B4DY09 | ILF2 | Interleukin enhancer-binding factor 2 |
| A0A087WWY6 | ILK | Integrin-linked protein kinase (Fragment) |
| M0QZX5 | ILVBL | Acetolactate synthase-like protein (Fragment) |
| H0Y4R1 | IMPDH2 | Inosine-5'-monophosphate dehydrogenase 2 (Fragment) |
| P07476 | IVL | Involucrin |
| Q9NS61-8 | KCNIP2 | Isoform 8 of Kv channel-interacting protein 2 |
| Q15058 | KIF14 | Kinesin-like protein KIF14 |
| Q15136 | KIN27 | Protein kinase A-alpha (Fragment) |
| Q5BQ95 | KLK13 | Kallikrein 13 splice variant 7 |
| Q86V93 | LOC100128510 | LOC100128510 protein |
| J3QKX9 | LOXHD1 | Lipoxygenase homology domain-containing protein 1 |
| P42704 | LRPPRC | Leucine-rich PPR motif-containing protein, mitochondrial |
| A6NM62 | LRRC53 | Leucine-rich repeat-containing protein 53 |
| B8ZZ12 | LUC7L | LUC7-like (S. cerevisiae) (Fragment) |
| A0A0A0MRB5 | MADD | MAP kinase-activating death domain protein |
| Q6ZN16-2 | MAP3K15 | Isoform 2 of Mitogen-activated protein kinase kinase kinase 15 |
| Q9Y2U5 | MAP3K2 | Mitogen-activated protein kinase kinase kinase 2 |
| D6RAC8 | MEPE | Matrix extracellular phosphoglycoprotein |
| P14174 | MIF | Macrophage migration inhibitory factor |
| Q9H8L6 | MMRN2 | Multimerin-2 |
| F2Z361 | MRPS14 | 28S ribosomal protein S14, mitochondrial |
| Q9BYN8 | MRPS26 | 28S ribosomal protein S26, mitochondrial |
| P82932 | MRPS6 | 28S ribosomal protein S6, mitochondrial |
| Q8N339 | MT1M | Metallothionein-1M |
| F5H2F4 | MTHFD1 | C-1-tetrahydrofolate synthase, cytoplasmic |
| H0YI04 | NAV3 | Neuron navigator 3 (Fragment) |
| Q9H094-2 | NBPF3 | Isoform 2 of Neuroblastoma breakpoint family member 3 |
| F5H0J3 | NDUFA9 | NADH dehydrogenase [ubiquinone] 1 alpha subcomplex subunit 9, mitochondrial |
| Q59FP8 | NEO1 | Neogenin (Fragment) |
| J3QSE9 | NLK | Serine/threonine-protein kinase NLK |
| K7EN42 | NMT1 | Glycylpeptide N-tetradecanoyltransferase 1 (Fragment) |
| P06748-3 | NPM1 | Isoform 3 of Nucleophosmin |
| O75607 | NPM3 | Nucleoplasmin-3 |
| B4DLR8 | NQO1 | NAD(P)H dehydrogenase [quinone] 1 |
| Q5T6F7 | NR5A1 | Steroidogenic factor 1 (Fragment) |
| A0A087WUD3 | OSTC | Oligosaccharyltransferase complex subunit OSTC |
| Q8TE49 | OTUD7A | OTU domain-containing protein 7A |
| C9JSL4 | P3H2 | Prolyl 3-hydroxylase 2 (Fragment) |
| E9PBS1 | PAICS | Multifunctional protein ADE2 (Fragment) |
| H0YMA5 | PCK | Phosphoenolpyruvate carboxykinase [GTP] |
| P20941-2 | PDC | Isoform 2 of Phosducin |
| P30101 | PDIA3 | Protein disulfide-isomerase A3 |
| P13667 | PDIA4 | Protein disulfide-isomerase A4 |
| K7EJ44 | PFN1 | Profilin |
| P53609-2 | PGGT1B | Isoform 2 of Geranylgeranyl transferase type-1 subunit beta |
| P00558-2 | PGK1 | Isoform 2 of Phosphoglycerate kinase 1 |
| F5GY37 | PHB2 | Prohibitin-2 |
| P78356-2 | PIP4K2B | Isoform 2 of Phosphatidylinositol 5-phosphate 4-kinase type-2 beta |
| M0QZK2 | PLD3 | Phospholipase D3 (Fragment) |
| Q8IY26 | PLPP6 | Phospholipid phosphatase 6 |
| H0YHI8 | PPP1R12A | Protein phosphatase 1 regulatory subunit 12A (Fragment) |
| P30048-2 | PRDX3 | Isoform 2 of Thioredoxin-dependent peroxide reductase, mitochondrial |
| P30041 | PRDX6 | Peroxiredoxin-6 |
| G3V0G2 | PRR5 | HCG2039433, isoform CRA_a |
| J3QQM1 | PSMC5 | 26S protease regulatory subunit 8 (Fragment) |
| J3QL24 | PYCR1 | Pyrroline-5-carboxylate reductase 1, mitochondrial (Fragment) |
| Q9Y2K5-2 | R3HDM2 | Isoform 2 of R3H domain-containing protein 2 |
| Q15042-4 | RAB3GAP1 | Isoform 3 of Rab3 GTPase-activating protein catalytic subunit |
| P51148 | RAB5C | Ras-related protein Rab-5C |
| C9JB90 | RAB6B | Ras-related protein Rab-6B (Fragment) |
| P78406 | RAE1 | mRNA export factor |
| K7EL35 | RHPN2 | Rhophilin-2 |
| Q9H1E1 | RNASE7 | Ribonuclease 7 |
| H7C123 | RPL10 | 60S ribosomal protein L10 (Fragment) |
| E7EPB3 | RPL14 | 60S ribosomal protein L14 |
| M0R3D6 | RPL18A | 60S ribosomal protein L18a (Fragment) |
| J3QL15 | RPL19 | Ribosomal protein L19 (Fragment) |
| G3V1B3 | RPL21 | 60S ribosomal protein L21 |
| K7EJT5 | RPL22 | 60S ribosomal protein L22 (Fragment) |
| J3KSS0 | RPL26 | 60S ribosomal protein L26 |
| K7EQQ9 | RPL27 | 60S ribosomal protein L27 |
| E9PLL6 | RPL27A | 60S ribosomal protein L27a |
| F2Z388 | RPL35 | 60S ribosomal protein L35 |
| J3KSP2 | RPL38 | 60S ribosomal protein L38 (Fragment) |
| P05386 | RPLP1 | 60S acidic ribosomal protein P1 |
| E9PS50 | RPS13 | 40S ribosomal protein S13 (Fragment) |
| M0QX76 | RPS16 | 40S ribosomal protein S16 (Fragment) |
| P62266 | RPS23 | 40S ribosomal protein S23 |
| E7ETK0 | RPS24 | 40S ribosomal protein S24 |
| Q5JNZ5 | RPS26P11 | Putative 40S ribosomal protein S26-like 1 |
| P62857 | RPS28 | 40S ribosomal protein S28 |
| B5MCT8 | RPS9 | 40S ribosomal protein S9 |
| F8WD59 | RPSA | 40S ribosomal protein SA (Fragment) |
| P31949 | S100A11 | Protein S100-A11 |
| Q9HCY8 | S100A14 | Protein S100-A14 |
| Q8WTV0-3 | SCARB1 | Isoform 2 of Scavenger receptor class B member 1 |
| F8W0Q0 | SCN8A | Sodium channel protein type 8 subunit alpha (Fragment) |
| Q58EX2-2 | SDK2 | Isoform 2 of Protein sidekick-2 |
| Q8NC51-4 | SERBP1 | Isoform 4 of Plasminogen activator inhibitor 1 RNA-binding protein |
| A0A024QZX5 | SERPINB6 | Serpin B6 |
| H0Y9U2 | SFPQ | Splicing factor, proline- and glutamine-rich (Fragment) |
| P34896-4 | SHMT1 | Isoform 4 of Serine hydroxymethyltransferase, cytosolic |
| E9PIB3 | SIPA1 | Signal-induced proliferation-associated protein 1 (Fragment) |
| E9PPP4 | SLC16A12 | Monocarboxylate transporter 12 (Fragment) |
| F5GZI0 | SLC3A2 | 4F2 cell-surface antigen heavy chain |
| P35610-2 | SOAT1 | Isoform 2 of Sterol O-acyltransferase 1 |
| F8VWW7 | SPRYD3 | SPRY domain-containing protein 3 |
| A0A0A6YYL2 | SULT1A4 | Sulfotransferase |
| Q7L0J3-2 | SV2A | Isoform 2 of Synaptic vesicle glycoprotein 2A |
| Q6ZMZ3-3 | SYNE3 | Isoform 3 of Nesprin-3 |
| Q8IV04 | TBC1D10C | Carabin |
| Q9UHD2 | TBK1 | Serine/threonine-protein kinase TBK1 |
| F8WE86 | TCN2 | Transcobalamin-2 |
| Q08188 | TGM3 | Protein-glutamine gamma-glutamyltransferase E |
| H3BVG9 | TIPIN | TIMELESS-interacting protein (Fragment) |
| D6RAA6 | TMEM33 | Transmembrane protein 33 (Fragment) |
| G3V155 | TMX2 | Thioredoxin domain containing 14, isoform CRA_a |
| Q9Y6Q6-5 | TNFRSF11A | Isoform 5 of Tumor necrosis factor receptor superfamily member 11A |
| H7C269 | TNRC6A | Trinucleotide repeat-containing gene 6A protein (Fragment) |
| P12270-2 | TPR | Isoform 2 of Nucleoprotein TPR |
| A0A0C4DFT0 | TRPM8 | Transient receptor potential cation channel subfamily M member 8 |
| F8WDA3 | TTC14 | Tetratricopeptide repeat protein 14 |
| P10599 | TXN | Thioredoxin |
| H0YN48 | UACA | Uveal autoantigen with coiled-coil domains and ankyrin repeats (Fragment) |
| A0A0A0MSL3 | UBE2V1 | Ubiquitin-conjugating enzyme E2 variant 1 |
| A0A0G2JMZ5 | UGT2B15 | UDP-glucuronosyltransferase |
| Q6PHR2-3 | ULK3 | Isoform 3 of Serine/threonine-protein kinase ULK3 |
| O15195-2 | VILL | Isoform 2 of Villin-like protein |
| A0A087WY55 | VTA1 | Chromosome 6 open reading frame 55, isoform CRA_b |
| O75717-2 | WDHD1 | Isoform 2 of WD repeat and HMG-box DNA-binding protein 1 |
| B1AHC9 | XRCC6 | X-ray repair cross-complementing protein 6 |
| Q6ZSB9-2 | ZBTB49 | Isoform 2 of Zinc finger and BTB domain-containing protein 49 |
| Q99676 | ZNF184 | Zinc finger protein 184 |

**Table 2. Nucleotide sequences of the primers for Real-time PCR.**

| **Gene** | **Forward** | **Reverse** |
| --- | --- | --- |
| mouse Trim21 | GGGAGGAGGTCACCTGTTCTA | GGCACTCGGGACATGAACTG |
| mouse Srebf1 | CACTTCTGGAGACATCGCAAAC | ATGGTAGACAACAGCCGCATC |
| mouse Pparγ | ATTCTGGCCCACCAACTTCGG | TGGAAGCCTGATGCTTTATCCCCA |
| mouse Fasn | TGGGTTCTAGCCAGCAGAGT | ACCACCAGAGACCGTTATGC |
| mouse Scd1 | TCTTCCTTATCATTGCCAACACCA | GCGTTGAGCACCAGAGTGTATCG |
| mouse Acc | AGGGTCAAGTGCTGCTCCA | GGCCAGTGCTATGCTGAGAT |
| mouse Pparα | TATTCGGCTGAAGCTGGTGTAC | CTGGCATTTGTTCCGGTTCT |
| mouse Acox1 | GTCTCCGTCATGAATCCCGA | TGCGATGCCAAATTCCCTCA |
| mouse Cd36 | GACTGGGACCATTGGTGATGA | AAGGCCATCTCTACCATGCC |
| mouse Pck1 | AGCATTCAACGCCAGGTTC | CGAGTCTGTCAGTTCAATACCAA |
| mouse β-actin | GATCATTGCTCCTCCTGAGC | ACTCCTGCTTGCTGATCCAC |

**Table 3. Nucleotide sequences of the primers for truncation PCR**

| **Gene** | **Forward** | **Reverse** |
| --- | --- | --- |
| TRIM-WT | GGATCTTCCAGAGATAAGCTTATGGCTTCAGCAGCACGC | CTGCCGTTCGACGATGAATTCATAGTCAGTGGATCCTTGTGATCCA |
| TRIM-RING^∆^ | GGATCTTCCAGAGATAAGCTTATGCAGCGCTTTCTGCTCAAG | CTGCCGTTCGACGATGAATTCATAGTCAGTGGATCCTTGTGATCCA |
| TRIM-SPRY^∆^ | GGATCTTCCAGAGATAAGCTTATGGCTTCAGCAGCACGC | CTGCCGTTCGACGATGAATTCTGCACATGTCCTCAGCATCTTC |

**Table 4. Nucleotide sequences of si-RNA.**

| **Gene** | **Forward** | **Reverse** |
| --- | --- | --- |
| Negative control | UUCUCCGAACGUGUCACGUTT | ACGUGACACGUUCGGAGAATT |
| si-TRIM21-148 | GAGGUCACAUGCCCUAUCUTT | AGAUAGGGCAUGUGACCUCTT |
| si-TRIM21-411 | CCUGUUCUGUGAGAAAGAUTT | AUCUUUCUCACAGAACAGGTT |
| si-TRIM21-1056 | GCAGAGCAUACCUGGAAAUTT | AUUUCCAGGUAUGCUCUGCTT |
